# The new genetic landscape of Alzheimer’s disease: from amyloid cascade to genetically driven synaptic failure hypothesis?

**DOI:** 10.1007/s00401-019-02004-0

**Published:** 2019-04-13

**Authors:** Pierre Dourlen, Devrim Kilinc, Nicolas Malmanche, Julien Chapuis, Jean-Charles Lambert

**Affiliations:** Unité INSERM 1167, RID-AGE-Risk Factors and Molecular Determinants of Aging-Related Diseases, Institut Pasteur de Lille, University of Lille, U1167-Excellence Laboratory LabEx DISTALZ, BP 245, 1, rue du professeur Calmette, 59019 Lille Cedex, France

## Abstract

A strong genetic predisposition (60–80% of attributable risk) is present in Alzheimer’s disease (AD). In view of this major genetic component, identification of the genetic risk factors has been a major objective in the AD field with the ultimate aim to better understand the pathological processes. In this review, we present how the genetic risk factors are involved in APP metabolism, β-amyloid peptide production, degradation, aggregation and toxicity, innate immunity, and Tau toxicity. In addition, on the basis of the new genetic landscape, resulting from the recent high-throughput genomic approaches and emerging neurobiological information, we propose an over-arching model in which the focal adhesion pathway and the related cell signalling are key elements in AD pathogenesis. The core of the focal adhesion pathway links the physiological functions of amyloid precursor protein and Tau with the pathophysiological processes they are involved in. This model includes several entry points, fitting with the different origins for the disease, and supports the notion that dysregulation of synaptic plasticity is a central node in AD. Notably, our interpretation of the latest data from genome wide association studies complements other hypotheses already developed in the AD field, i.e., amyloid cascade, cellular phase or propagation hypotheses. Genetically driven synaptic failure hypothesis will need to be further tested experimentally within the general AD framework.

**Electronic supplementary material:**

The online version of this article (10.1007/s00401-019-02004-0) contains supplementary material, which is available to authorized users.

## Introduction

Alzheimer’s disease (AD) is the most common neurodegenerative disorder and constitutes a major public health problem worldwide (with 35.6 million sufferers). AD causes memory loss and cognitive impairment, which are invariant early signs of the disease. Hippocampal atrophy due to neuronal death is one of the earliest hallmarks of AD. Preceding neuronal death, synaptic dysfunction and synapse loss have been observed in post-mortem AD brains and are correlated with cognitive decline [28]. AD is characterised by the coexistence of two main pathological lesions: (1) intraneuronal neurofibrillary tangles composed of abnormally modified Tau proteins and (2) parenchymal amyloid deposits centred around β-amyloid (Aβ) peptides. The discovery of rare mutations in *APP*, *PSEN1* and *PSEN2* causing autosomal dominant forms of the disease gave rise to the amyloid cascade hypothesis, which radically changed our understanding of AD [46]. Systematic association of pathogenic mutations with altered amyloid precursor protein (APP) metabolism and with the overproduction of longer Aβ peptides in particular—which are believed to be more neurotoxic—suggest that these peptides are at the heart of the disease process. Overproduction of these neurotoxic peptides may lead to (or accentuate) the neuron-to-neuron propagation of Tau pathology by a still unknown mechanism [20]. However, since monogenic forms represent less than 1% of all AD cases, crucial questions can be raised: (1) is the amyloid cascade hypothesis central to all forms of AD? (2) Are other pathophysiological processes involved in late-onset non-Mendelian forms of AD?

Within this context, understanding the genetics of the most common forms of AD would improve our knowledge of the underlying pathophysiological processes. Indeed, genetic risk factors account for up to 80% of attributable risk in these common forms [30]. One can thus argue that the vast majority of the AD pathophysiological pathways are driven by or include genetic determinants. Accordingly, describing these genetic determinants should improve our understanding of the fundamental disease processes. In this review, we present the latest advances in AD genetics and in the post-genome wide association studies (GWASs) era, and discuss how these information might change our understanding of the AD pathophysiological processes.

## Genetic landscape of AD

Over a 16-year period (from 1993 to 2009), the Apolipoprotein E (*APOE*) gene was the only genetic risk factor identified for AD. The association of the *APOE* ε4 allele with AD risk has been repeatedly demonstrated, whereas the ε2 allele was associated with a protective effect [71]. *APOE* is estimated to account for 20% of the AD attributable risk, and the *APOE*-associated risk is similar to those identified for major genes in other Mendelian diseases, such as *BRCA1* in breast cancer [31]. Despite testing more than 500 candidate genes for their association with AD risk [79], we had to wait for the development of GWAS to advance the AD genetics field: since the seminal GWAS papers published in 2009 [47, 83], more than 30 loci of interest have been identified to be associated with AD risk (Suppl. Table) [50, 59, 67, 76, 84, 97, 116, 120]. Additionally, several analyses of higher complexity have been developed based on these GWAS datasets, allowing the identification of additional genes of interest (Suppl. Table) [10, 41, 67, 82].

Over the same period, next generation sequencing (NGS) has been developed and became applicable to multifactorial diseases due to remarkable cost decreases. NGS allowed the identification of rare variants in *TREM2* as major genetic risk factors for AD [39, 65]. In addition, these methodologies demonstrated that both *SORL1* and *ABCA7* genes—already detected by GWAS—carried numerous loss-of-function variants leading to strong increases in AD risk [9, 98, 128]. Finally, the Alzheimer’s disease sequencing project (ADSP) recently published its first results based on whole exome sequencing analysis, encompassing more than 5000 AD cases and controls, and reported two new candidate genes, *IGHG3* and *ZNF655* (Suppl. Table) [11].

**Fig. 1:**
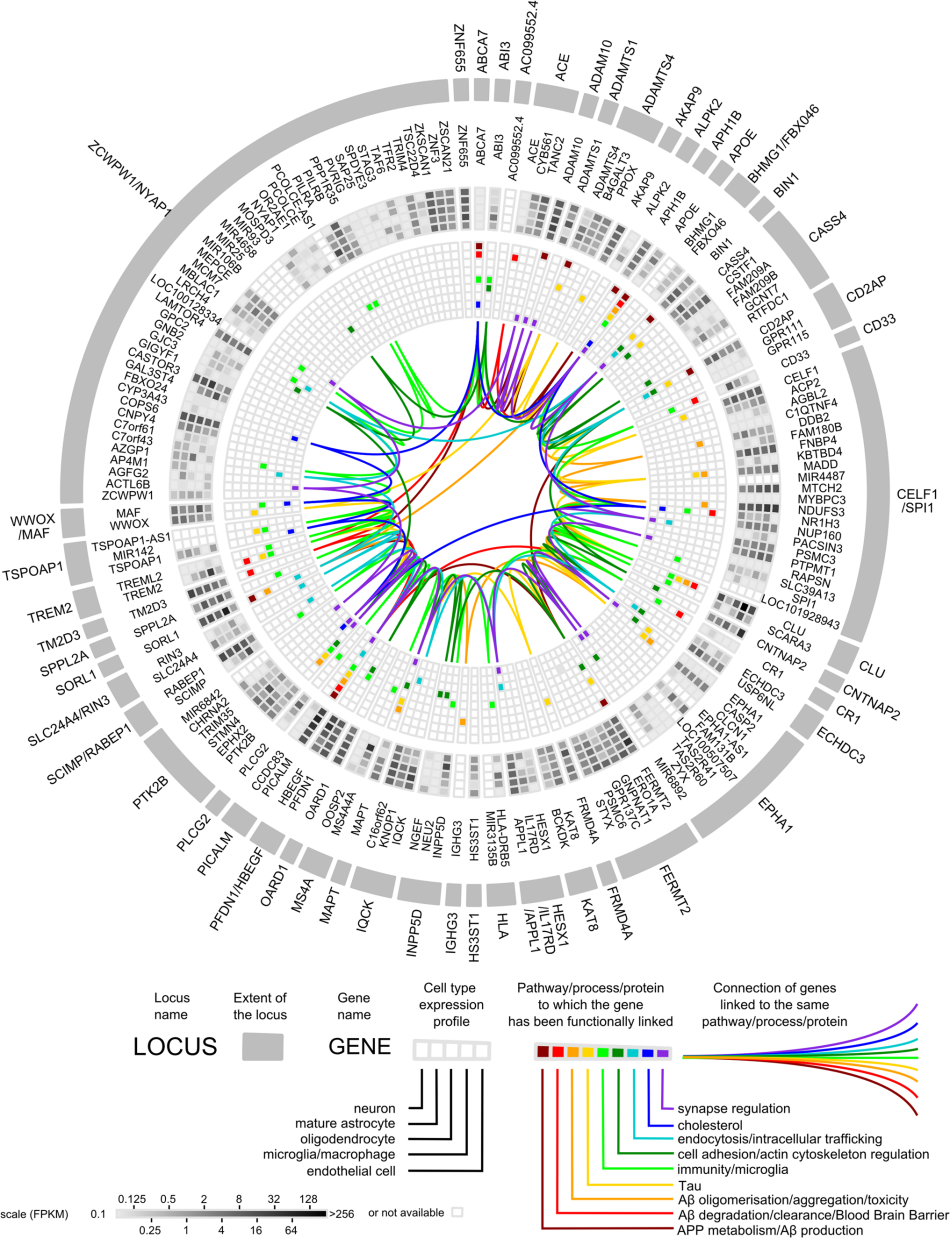
Circular diagram of AD genetic risk factors. The diagram shows (from outside to inside): (1) genomic loci in alphabetical order; (2) genes therein; (3) expression profiles of these genes in different cell types of the brain (grayscale); and (4) the pathways/processes/proteins to which these genes have been functionally linked (colour). Details of the functional studies supporting these linkages are available in Suppl. Table. Expression profiles were extracted from [155] (*FPKM* fragments per kilobase of transcript sequence per million mapped fragments). The circlize package of the *R* software (http://www.r-project.org/) was used to generate the diagram [38]

Together, these genetic analyses have pointed out more than 45 genes/loci associated with the risk of developing AD even if it is estimated that a large part of the genetic component of AD is still unknown [103]. In addition, it is essential to keep in mind that for some loci, it is difficult—if not impossible—to determine the gene responsible for the observed association, due to the presence of multiple genes in the locus and the complex linkage disequilibrium patterns involving the sentinel single-nucleotide polymorphism. Nevertheless, it is now possible to start drawing a general landscape of what the genetics is telling us. According to multiple post-genomic analyses performed in cellular and animal models, this landscape already appears to be highly complex (Fig. 1).

## The post-GWAS era and the amyloid cascade hypothesis

**Fig. 2:**
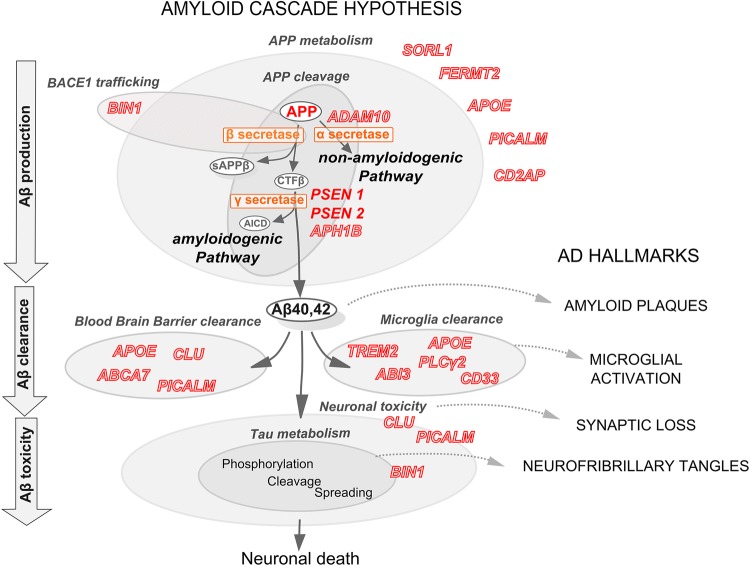
Amyloid cascade hypothesis and genetic risk factors of AD. Autosomal dominant mutations that cause early onset familial AD (in *APP*, *PSEN1* and *PSEN2*) gave rise to the amyloid cascade hypothesis, which aims to link amyloid plaques and neurofibrillary tangles, the two classical AD hallmarks. Involvement of the genetic risk factors of late-onset AD in APP metabolism and in Aβ clearance through the blood brain barrier or microglia supports this hypothesis. Soluble forms of Aβ may be inducing neurotoxicity through modifying Tau metabolism, leading to neurofibrillary tangle formation and neuronal death. Recent GWAS-defined genes that modulate Tau toxicity may be involved in Aβ-induced neurotoxicity through mechanisms that are yet to be identified

As previously mentioned, the identification of familial AD-linked mutations in the genes for APP, PS1, and PS2, associated with the dysregulation of Aβ peptide production has suggested that APP metabolism is at the heart of the disease process. Three main proteases (α-, β- and γ-secretases) are involved in APP processing through (1) the amyloidogenic pathway (β- and γ-secretases), which promotes Aβ production, and (2) the non-amyloidogenic pathway (α- and γ-secretases), which prevents Aβ production by cleaving APP within the Aβ sequence. In addition to secretase activity, APP trafficking by the secretory pathway is also an essential factor in APP metabolism. APP is first matured in the endoplasmic reticulum and the Golgi apparatus, and then transported to the cell surface. Alternatively, APP can end up in the lysosomal pathway and undergo proteolytic degradation [115]. Naturally, it has been rapidly proposed that GWAS-defined genes may have a role in APP metabolism, especially since gene-set enrichment analyses performed on GWAS datasets identified specific pathophysiological pathways—among others—involved in APP processing and in the regulation of endocytosis, which is central for APP metabolism (Fig. 2) [63, 76, 81].

### APP metabolism and Aβ peptide production

Very recent GWASs have strengthened the importance of APP metabolism by directly pointing out two of its main actors as AD genetic risk factors: ADAM10, which carries out the main α-secretase activity in the brain, and APH1B, which is part of the γ-secretase complex [59, 76].

Based on pathway enrichment analyses, three GWAS-defined genes linked with endocytosis (*BIN1*, *CD2AP* and *PICALM*) have been proposed to modulate APP metabolism and the subsequent increase in Aβ production: the best characterised function of BIN1 is its role in endocytosis, where it interacts with several proteins associated with clathrin-coated pits and endosomal sorting [99]. BIN1 depletion was associated with an increase in intracellular Aβ_42_, potentially through the regulation of the β-secretase (BACE1) trafficking and accumulation in early endosomes [91, 137]. CD2AP, which is also a regulator of endosome trafficking, has been reported to control Aβ production in neurons. CD2AP depletion leads to the trapping of APP in early endosomes and the subsequent increase in Aβ [137]. However, despite these effects on Aβ production in vitro, deleting a single *BIN1* or *CD2AP* allele did not modify amyloid deposition in mouse models of AD [2, 87]. Nevertheless, it is worth noting that abnormal accumulation of insoluble BIN1 has been detected in the vicinity of amyloid plaques in different mouse models [107]. Finally, PICALM, which is also directly involved in endocytosis, has been linked to APP metabolism: PICALM underexpression decreases the levels of several APP catabolites, such as intracellular β-C-terminal fragment (β-CTF) and secreted sAPPβ, which has been attributed to its role in clathrin-mediated endocytosis [134]. Accordingly, PICALM underexpression results in reduced APP internalisation and Aβ generation [148]. As opposed to BIN1 and CD2AP, PICALM knockdown and overexpression, respectively, decreased and increased amyloid plaque loads in the hippocampi of 6-month-old APP/PS1 mice [148]. Alternatively, PICALM has been proposed to participate in the APP-CTF degradation through autophagy [135].

In addition to these three genes that are known to be directly involved in endocytosis, ABCA7 underexpression induced faster APP endocytosis, consistent with increased Aβ production in vitro and accelerated the amyloid pathology in young transgenic mice [111]. Finally, ApoE4 has been described as a potential enhancer of amyloid peptide production through stimulating APP endocytosis and metabolism, both in vitro and in vivo [48, 49].

Apart from the genes susceptible to control APP/BACE1 availability in early endosomes through the modulation of endocytosis, other GWAS-defined genes have been shown to modulate APP metabolism through affecting APP sorting from endosomes. Kindlin-2 (product of *FERMT2*) underexpression has been described to promote the recycling of full-length, mature APP from early/late endosomes into the plasma membrane. This may thus favour Aβ production by increasing the pool of full-length APP likely to be cleaved by α-, β- and γ-secretases, rather than to be degraded in lysosomes [17]. SORL1 underexpression has also been associated with an increase in Aβ production by blocking the redirection of endocytosed APP to the trans-Golgi network, which reduces APP processing in endosomes [113, 144, 152]. Alternatively, it has been suggested that SORL1 loss-of-function may decrease the degradation of intracellular Aβ by lysosomes [13].

In conclusion, post-GWAS analyses seem to place numerous GWAS-defined genes in the APP metabolism landscape, deeming its importance in late-onset AD. Remarkably, while the dysregulation of APP metabolism is believed to mainly occur in neurons, ADAMTS4, exclusively expressed in oligodendrocytes, has been recently reported to produce strongly aggregating Aβ forms [139]. In fact, ADAMTS4 has been very recently reported as an AD genetic risk factor [59], suggesting that multiple cell types participate in Aβ production in AD.

### Aβ peptide degradation/clearance

Beyond Aβ production, which is of course a major factor controlling Aβ bioavailability and potential toxicity, modulation of Aβ aggregation and/or degradation/clearance has been proposed to be essential for Aβ toxicity. The initial GWAS data rapidly led to a reappraisal of the amyloid cascade hypothesis based on Aβ accumulation and toxicity in both familial and early onset forms of AD where Aβ peptides rapidly accumulate due to their accelerated overproduction (e.g., associated with mutations in *APP*, *PSEN1* and *PSEN2*), and in late-onset forms, due to a slight, insidious Aβ clearance impairment (associated with several GWAS-defined genes) [80].

In this view, consistent evidence strongly suggest that ApoE regulates extracellular Aβ clearance in the brain [71]. In animal models, Aβ–ApoE2 and Aβ–ApoE3 complexes are cleared by the blood brain barrier (BBB) at a substantially higher rate than Aβ–ApoE4 complexes [23]. ApoE binds to a group of structurally related proteins known as the low density lipoprotein receptor (LDLR) family. LDLR overexpression dramatically enhanced Aβ clearance from the brain’s extracellular fluid and reduced Aβ aggregation [72]. CLU, which is one of the most abundantly expressed apolipoproteins in the central nervous system (like ApoE) [106], participates in Aβ clearance, principally of the highly pathogenic Aβ_42_ peptide, from the brain across the BBB [24]. More recently, PICALM has been described in vitro and in vivo to participate to Aβ transcytosis and clearance at the BBB through clathrin-dependent internalisation of Aβ after its binding to the LDLR-related protein-1 [157]. ABCA7 is also potentially involved in amyloid clearance at the BBB [78]. Finally, a peripheral Aβ clearance mechanism involving CR1 in human erythrocytes has been proposed to be impaired in AD cases [105].

However, beyond these potential BBB and peripheral clearance mechanisms, microglia dysfunction has also been suspected to be involved in AD through modulating Aβ aggregation/degradation [45]. Indeed, several genomic analyses suggest the involvement of microglia in AD: (1) the first reports on pathway enrichment analyses indicated the involvement of innate immunity in AD [81]; (2) as previously mentioned, these analyses highlighted the regulation of endocytosis (which is essential for phagocytosis) [64, 81]; (3) a large part of GWAS-define genes are expressed in microglia [45] (Fig. 1); and (4) a major genetic discovery indicated that non-synonymous variants in *TREM2*, *ABI3* and *PLCγ2* were associated with AD risk [40, 65, 120]. In the brain, these three genes are almost exclusively expressed in microglia and participate in the same protein–protein interaction network [120]. In addition *SPI1* carrying a common variant associated with a decreased AD risk encodes the PU.1 transcription factor, which is a key player in microglia development [56].

Apart from ApoE [118], TREM2 is the most studied genetic risk factor in the context of the microglia-dependent pathophysiological process in AD. TREM2 has been proposed to be protective, since the AD-associated mutations (R47H, R62H) likely impair its physiological functions at two levels: (1) phagocytosis and clearance of Aβ peptides and (2) compaction of amyloid plaques and barrier formation. In AD-like transgenic mice models, TREM2 knockdown is associated with increased amyloid loads [60, 141]. TREM2-deficient microglia show reduced uptake of Aβ complex in vitro and less Aβ internalisation in vivo [150, 154]. Importantly, microglial uptake of Aβ is more efficient when TREM2 forms complexes with lipoproteins such as ApoE or CLU [132, 150] and TREM2–lipoprotein interaction is impaired by R47H and R62H mutations [3, 4]. Furthermore, microglia have been recently suggested to form a protective barrier around amyloid deposits by compacting amyloid fibrils into a potentially less toxic form [154]. This protective mechanism appears to be less efficient in AD-like models underexpressing TREM2. In addition, in the brains of humans with R47H TREM2 mutation, microglia present a markedly reduced ability to envelop amyloid deposits [154]. Again, one can suspect a protective mechanism linking ApoE and TREM2, since ApoE is also involved in the compaction of the protofibrillary Aβ into dense plaques [5, 6].

Among other GWAS-defined genes, very few have been analysed in the microglia context, and only CD33 has been described to inhibit Aβ uptake by microglia [37]. In conclusion, there is no longer any doubt that microglia participate to AD pathogenesis, potentially reinforcing the amyloid cascade hypothesis through Aβ peptide clearance and or/compaction.

### Aβ peptide toxicity

According to the amyloid cascade hypothesis, Aβ peptides are neurotoxic and supposed to lead to or accentuate neuron-to-neuron propagation of Tau pathology—leading to neuronal death—by a still unknown mechanism [20]. Beyond ApoE, which has been reported as a potential modulator of Aβ toxicity (see reviews [51, 131]), little has been done to determine whether other GWAS-defined genes are involved in Aβ toxicity. RNAi knockdown of *amph*-*1* and *unc*-*11* (orthologs of *BIN1* and *PICALM*, respectively) in transgenic *C. elegans* over-expressing Aβ_42_ in body wall muscle cells significantly delayed paralysis due to Aβ_42_ toxicity [95]. However, no molecular mechanism has been proposed so far to explain these findings. CLU has also been associated with the regulation of Aβ toxicity, where its knockdown leads to reduced toxicity through the involvement of Wnt-PCP-JNK pathway [70]. In conclusion, Aβ toxicity needs to be explored in the view of the new genetic landscape.

## Beyond Aβ peptides and the APP metabolism

Although the data described above clearly support the amyloid cascade hypothesis, it is necessary to keep in mind that the biological characterization of GWAS-defined genes was initially done by assessing their impacts on models specifically developed for this hypothesis. There is, therefore, a significant bias, potentially leading to tautological reasoning and a priori exclusion of other, potentially important pathophysiological pathways. This is especially true since most GWAS-defined genes are involved in major cell signalling pathways and have pleiotropic functions, which are poorly evaluated using in vitro and in vivo models with specific read-outs. In addition, the functions of these genes are often not fully understood, and some of these unknown functions may be essential for their involvement in AD pathophysiology. This is also true for the most recognized actors of AD pathophysiology, such as APP or Tau. For instance, our knowledge of APP’s physiological functions are still very fragmented and often revolve about Aβ toxicity, while ignoring the potential functions of APP other than producing Aβ. Furthermore, APP metabolism produces other catabolites with roles potentially as important as that of Aβ. Our knowledge on the role of Tau in AD is also fragmented. We are thus unable to explain how Aβ peptides—despite being a part of the amyloid cascade—may participate in the neuron-to-neuron propagation of Tau pathology [20]. In conclusion, the impact of GWAS-defined genes on AD pathophysiology has so far been mostly studied in the context of the amyloid cascade hypothesis. This is clearly a limitation considering the increasing number of new genetic risk factors, which may potentially lead to the identification of not only Aβ toxicity-dependent mechanisms, but also Aβ-independent mechanisms (potentially linked to Tau).

### Post-GWAS analyses and Tau

Although GWAS-defined genes have not been extensively studied in the context of Tau pathology, it appears that several of them may interfere with Tau in AD. Among them, the largest amount of compiled evidence is on *BIN1*, which has been identified as the first AD genetic risk factor linked to Tau pathology. Loss- and gain-of-function of the *Drosophila* ortholog of *BIN1* modified the rough eye phenotype induced by human Tau expression [18]. In addition, a functional risk variant of *BIN1* has been associated with Tau loads (but not Aβ loads) in AD brains [18]. Furthermore, human BIN1 directly binds Tau and this interaction in neurons depends on the level of BIN1 expression, as well as the phosphorylation statuses of both BIN1 and Tau [85, 89, 110, 124]. Moreover, human BIN1 overexpression in a mouse model of tauopathy has been shown to increase BIN1–Tau interaction in the neuronal network and to rescue long-term memory deficits and Tau somatic inclusions induced by human Tau overexpression [110].

The *Drosophila* rough eye phenotype has also been used to perform genetic screens to systematically test GWAS-defined genes as potential modulators of Tau toxicity [25]. Beyond *BIN1*, this approach identified *p130CAS*, *Fak*, *Eph*, *Rab3*-*GEF*, *cindr*, *Fit1, Aret* and *Rhea* respective orthologs of *CASS4*, *PTK2B*, *EPHA1*, *MADD*, *CD2AP*, *FERMT2, CELF1* and *TLN2* [26, 42, 119]. Pyk2 (product of *PTK2B*) has been further shown to accumulate early in the somata of neurons exhibiting Tau pathology in AD patients and in a mouse model [26]. In addition, being a tyrosine kinase, Pyk2 has been shown to directly phosphorylate Tau [86]. In conclusion, a non-negligible part of GWAS-defined genes are also potentially involved in pathophysiological processes related to Tau.

### Post-GWAS functional screens point towards the core of the focal adhesion pathway

Remarkably, a large part of these GWAS-defined genes interacting with Tau pathology take part in the focal adhesion complex, mainly downstream of integrins (Suppl. Table 1; Fig. 2). Pyk2 and CASS4 are members of the FAK and CAS families of proteins that interact physically and mediate cell adhesion signalling [8]. CD2AP is involved in cell–cell adhesion, regulates the actin cytoskeleton and physically interacts with p130CAS (CASS1) [61, 74]. Kindlin-2 is a focal adhesion (FA) protein involved in integrin activation [77] and is also able to directly bind filamentous actin (F-actin) to modulate its organisation [12]. TLN2 is a cytoplasmic adapter protein essential for integrin-mediated cell adhesion to the extracellular matrix [35]. Finally, without being previously described as part of the core of the FA pathway, BIN1 physically interacts with FA kinase (FAK), which presents strong homology with Pyk2 [90]. In addition, BIN1 binds to F-actin and microtubule-binding protein CLIP170 in non-neuronal cells [21] and directly remodels actin dynamics through its BAR domain [27].

**Fig. 3:**
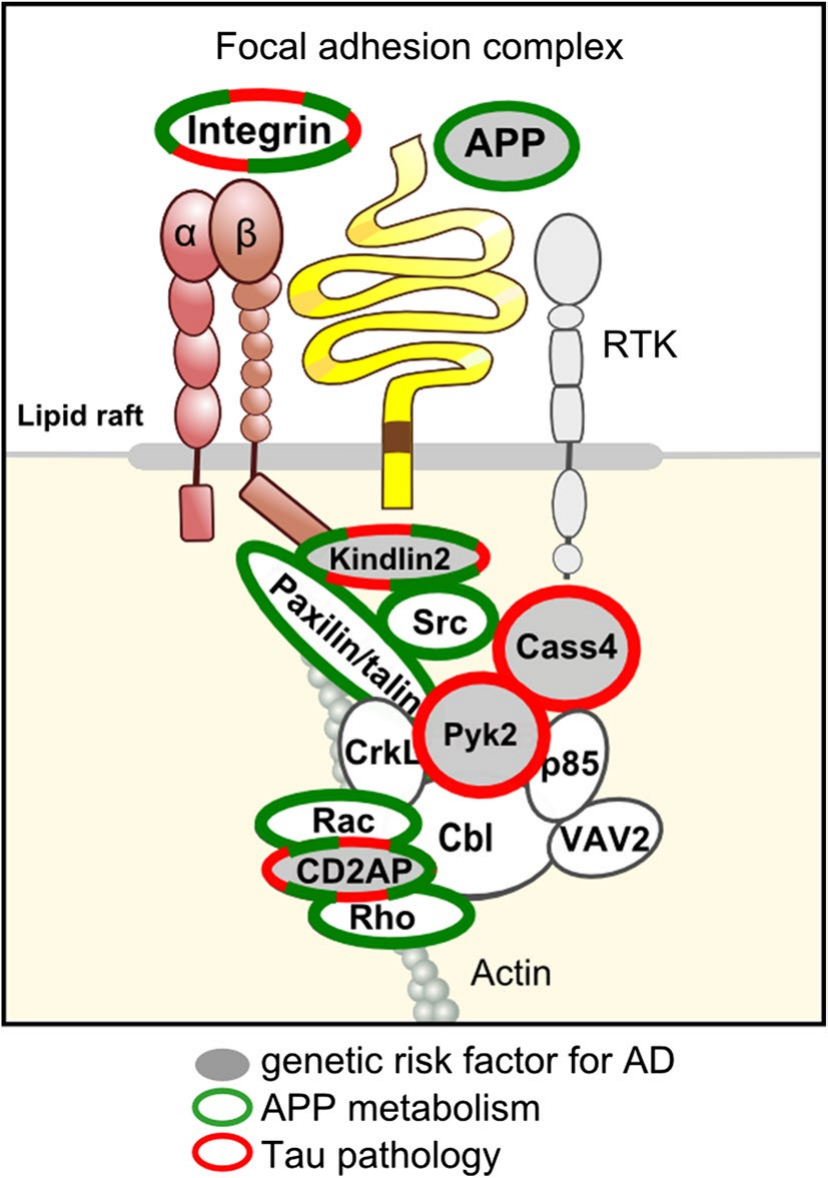
Interactions between integrin, APP and receptor tyrosine kinases (RTKs) at the cell surface modulate cell adhesion. The GWAS-defined genes *FERMT2*, *Cass4*, *PTK2B* and *CD2AP*, recently identified for their roles in APP metabolism (in green) and/or Tau pathology (in red), are involved in the focal adhesion complex, which regulates several downstream cell signalling pathways as well as the actin cytoskeleton. This observation supports the concept that the focal adhesion core, together with its related downstream pathways, may be an important actor in the AD process

Importantly, high-content screening also identified numerous FA pathway members—including Kindlin-2—as modulators of APP metabolism, highlighting the relevance of the core FA proteins to the AD process [17]. Therefore, despite not been considered initially, systematically screening AD risk factors for their capacity to modulate Tau toxicity and APP metabolism (in *Drosophila* and in cellular models) highlighted the core of the FA pathway for its potential involvement in the molecular mechanisms of AD pathogenesis (Fig. 3).

### Focal adhesion pathway, GWAS-defined genes and synapse dynamics

Among numerous biological functions, the FA pathway can modulate F-actin dynamics through regulating multiple actin-binding proteins, e.g., cortactin, profilin, drebrin or cofilin, and controls the shape of the dendritic spine [125]. The integrin-dependent FA pathway has been subsequently involved in synaptic density and activity through spine shape, stability and signalling machinery therein [54]. Since synaptic dysfunction and loss is one of the very early hallmarks of AD correlated with early cognitive decline, the high number of genetic determinants situated in a pathway that is heavily involved in synapse dynamics naturally calls for the assessment of the potential roles of these genes in synapse dynamics.

To date, only a few GWAS-defined genes have been studied in the context of synapses. Nevertheless, several lines of evidence seem to emerge to support their potential involvement in synapse dynamics [69]. As previously mentioned, products of several GWAS-defined genes are already known to regulate the actin cytoskeleton, e.g., CD2AP, Kindlin-2 and BIN1. Besides regulating the F-actin network, several of these GWAS-defined genes are directly involved in synapses in the physiological context. Our preliminary data concerning Kindlin-2 indicate that this protein is localized in both pre- and post-synaptic compartments (unpublished observations). Pyk2 is also localised to synapses and decreased Pyk2 expression in mice has recently been associated with alterations in dendritic spine density and in *N*-methyl-d-aspartate receptors (NMDAR) and PSD-95 distribution in spines [33]. Consistently, Pyk2 was involved in long-term potentiation (LTP) through the regulation of Src in an activity-dependent fashion [57], and in long-term depression (LTD) [55, 109]. Finally, increased expression of BIN1 in mice has also been associated with alterations in dendritic spine density and in LTP dysregulation [22].

### APP’s role in synaptic function and AD-related synaptic dysfunction

A growing body of evidence also suggests a physiological role for APP in synaptic functions [96], potentially through cell adhesion. During development, APP is enriched in axonal growth cones and acts as a co-receptor for guidance cues through its interaction with the extracellular matrix [122, 123]. Accordingly, APP–integrin interaction is required for neurite outgrowth and contact guidance [123, 153]. After differentiation, APP acts as a synaptic adhesion molecule required at both presynaptic and postsynaptic membranes for correct synapse patterning [142]. Inhibition of APP shedding strongly enhances cell adhesion and synaptogenic activity [127]. The APP intracellular domain (AICD) is required for normal synaptic morphology and synaptic plasticity, suggesting the involvement of other interactors for proper synapse formation [75]. Thirty-five percent decrease in spine density has been observed in neurons derived from APP^−/−^ mice, which was partially rescued by sAPP-α conditioned medium [136]. In this context, modified APP cleavage/degradation may modulate the membranous pool of APP or its metabolic products (e.g., AICD, APPs-α) and impair APP function in cell adhesion and synaptic plasticity [117]. These APP functions appear to be related to the FA pathway: integrin and APP share a number of interactors, including Fe65, a cytoplasmic adaptor protein that can simultaneously bind two NPXY motifs (carried by both integrins and APP) to form tripartite complexes [108]. Accordingly, APP can be recruited into a multimeric complex with β1-integrin. Altogether, these observations imply that cell surface APP modulates integrin signalling by recruiting different intracellular partners. Notably, targeting cell surface APP with antibodies induces neuronal injury through dysregulation of FAK and paxillin phosphorylation [149].

Beyond their impact on Aβ production (as described earlier), AD genetic risk factors may disrupt APP function and/or its interaction with the FA pathway. For example, Kindlin-2 expression is required for the recruitment and activation of FAK to trigger integrin signalling [100, 133], but Kindlin-2 is also a modulator of cell surface APP levels [17]. Interestingly, SORL1 has also been described to shuttle APP from early endosomes back to the Golgi apparatus and to subsequently impair its trafficking to the cell surface [1, 114]. Finally, the α-secretase ADAM10 has been functionally associated with several integrins [62, 121]. In conclusion, reports of numerous genetic risk factors involved in the core of the FA pathway point to a potential impairment of APP functions in cell adhesion and synaptic plasticity in AD, potentially independent of Aβ peptide production.

### Aβ toxicity and synaptic dysfunction through FA

FAs have also been established as regulators of Aβ signalling [15]; however, the mechanistic details of the regulatory mechanisms are poorly understood, as are their potential links to the genetic component of the AD. Integrin signalling has been described to mediate Aβ-induced neurotoxicity in hippocampal neurons via FAK signalling [44]. Activation of integrin signalling by Aβ fibrils has been associated with enhanced NMDAR sensitivity [138] and inhibition of LTP [140]. In addition, aberrant activation of the FA pathway may mediate Aβ fibril-induced neuronal dystrophy [36]. Aβ fibril-induced dystrophy requires the activation of FA pathway and the formation of aberrant FAs, suggesting a mechanism of maladaptive plasticity in AD. More specifically, rapid tyrosine phosphorylation of neuronal proteins including Tau and FAK can be observed in response to Aβ exposure involving Src family kinases [143]. It has been also proposed that the scaffolding protein RanBP9, which exhibits an overall increase in AD brains, simultaneously promotes Aβ generation and FA disruption by accelerating APP and β1-integrin endocytosis [145]. RanBP9 activates/dephosphorylates cofilin, a key regulator of actin dynamics [146]. Formation of cofilin–actin rods in distal dendrites is detrimental for synapses [7, 19] and cofilin–actin rod pathology has been observed in the brains of post-mortem AD patients [101] and in animal models of AD [7]. Together, these results implicate the integrin–cofilin pathway to be critical in synaptic dysfunction in AD [36, 147]. Cumulatively, these studies strongly suggest that AD is accompanied by an impairment of the FA pathway—potentially linked to Aβ toxicity—leading to altered synapse dynamics and eventually to synapse loss.

### Tau in synaptic function and AD-related synaptic dysfunction

The potential involvement of GWAS-defined genes related to Tau toxicity in synapse dynamics and in AD synaptic pathology would be supported if Tau itself had a role in synaptic function. Interestingly, several recent studies suggest that Tau may directly regulate synaptic function and plasticity: in healthy neurons, Tau is observed in the dendritic shaft as well as in pre- and post-synaptic structures [92, 94, 156]. Post-synaptic Tau has been demonstrated to be crucial for LTD [73]. Importantly, Tau’s role in regulating synapses does not appear to be redundant or functionally compensated in Tau knockout mice, suggesting a physiological role for Tau in synapses [151]. While little is known about the cellular mechanisms linking Tau with synaptic function, Tau activity, localization and function in synapses appear to be phosphorylation-dependent [58].

Whereas the physiological roles of Tau in synapses have recently been identified (and require further investigation), numerous studies have already established Tau as a mediator of AD-related synaptic deficits (for a review see [126]). Indeed, Tau has been implicated in the synaptotoxicity induced by Aβ exposure [102]. In this context, Tau is partially re-localized to the post-synaptic domain where it is thought to promote Aβ toxicity in pathological conditions by modifying NMDAR activation [53]. Aβ-induced dendritic spine loss [88] and neuronal death [130] depend on Tau phosphorylation. Aβ exposure modifies Tau’s phosphorylation status and prevents it from relocalizing away from the post-synaptic domain following synaptic activation [29]. Moreover, Tau–Fyn interaction in synapses has been shown to be required to induce pathology in mice over-expressing APP, where reduced Tau levels decrease excitotoxicity and reduced Fyn levels prevent Aβ toxicity [104].

Very few data are currently available linking GWAS-defined genes with Tau pathology and with synapses. The most promising results are for Pyk2 which may be participating in the Fyn–Tau crosstalk. As mentioned earlier, we were able to detect a genetic interaction between Pyk2 and Tau [26]. Pyk2 has been reported to mediate GSK-3 activation and subsequent Tau phosphorylation [112]. In addition, Pyk2 is able to activate Fyn [16], which, in turn, modulates Pyk2 activation in mouse brains [43]. This reciprocal regulation is further supported by the observation that Aβ-induced Fyn signalling is associated with downstream Pyk2 phosphorylation [68]. Finally, Pyk2 has recently been shown to mediate Aβ-dependent synapse loss [109]. In conclusion, based on the limited amount of data currently available, we propose that emerging evidence supports that Tau function is crucial for normal synaptic physiology and it may be dysregulated in AD potentially through interaction with genetic risk factors in an Aβ-dependent or Aβ-independent manner.

## From an Aβ-centred to a synapse-centred hypothesis?

Over one decade, GWASs and high-throughput sequencing approaches have fully revolutionized our knowledge of the genetics of AD. In this new genetic landscape, as mentioned earlier, AD appears as a complex, multifactorial disease resulting from a complex crosstalk between different pathophysiological processes. Importantly, the amyloid cascade hypothesis appears to be supported by this new genetic landscape, which additionally implicates microglia to have a major role in common AD forms potentially through Aβ peptide clearance. However, these genetic data also highlight the core FA and its related downstream pathways to be potentially central to AD pathophysiology. Interestingly, this particular pathway was not predicted by in silico gene ontology enrichment analyses conducted on GWAS-based gene lists; it rather emerged on the basis of in vivo and in vitro biological evidence. Notably, several functions of GWAS-defined genes were unknown before this evidence was obtained.

**Fig. 4:**
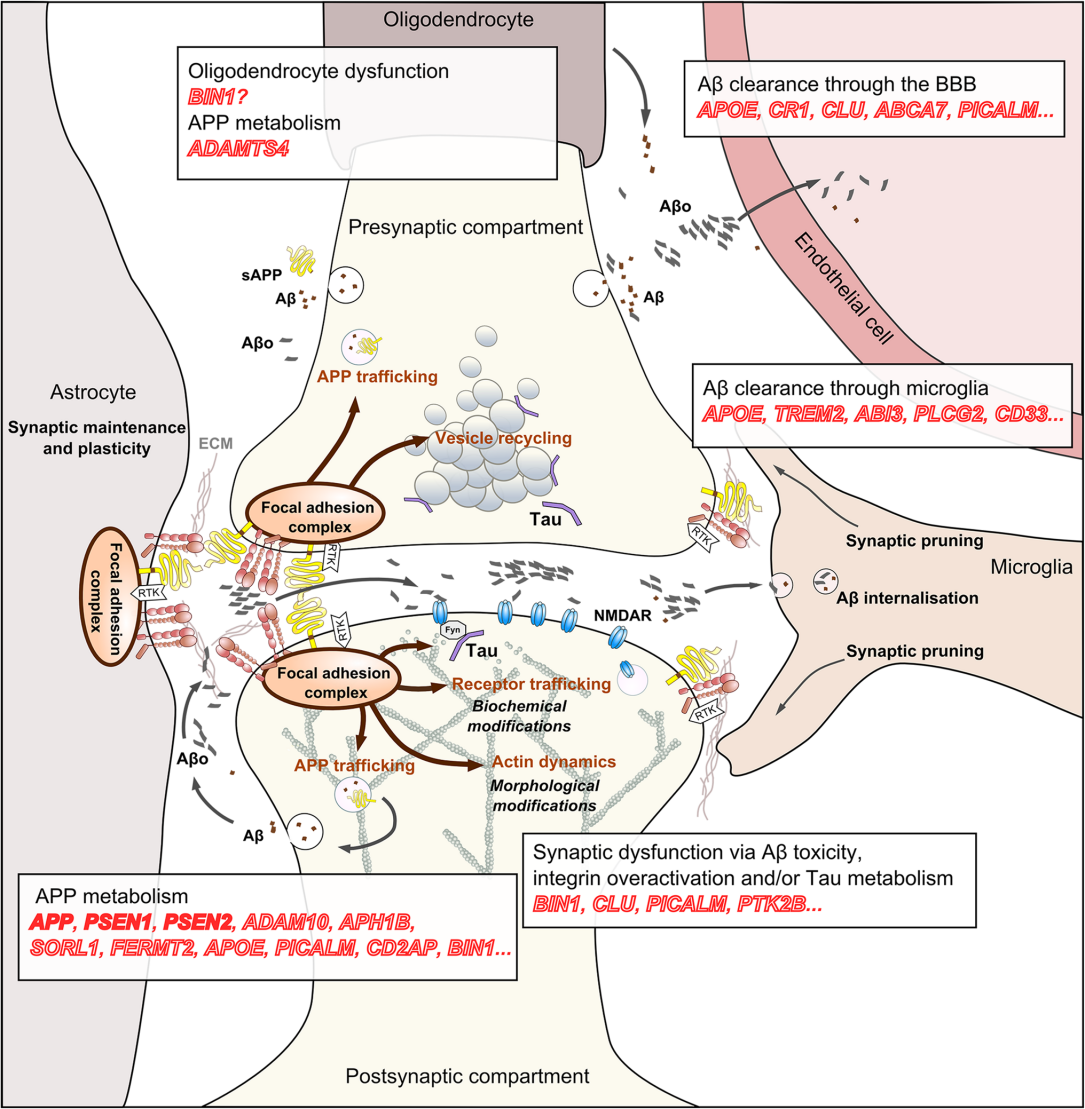
Genetic risk factors and synapse dysfunction in AD pathogenesis. Regulation of the focal adhesion pathway plays central roles in synaptic plasticity (synaptic maintenance, actin cytoskeleton remodelling, vesicle, and receptor cycling). Dysfunction of downstream cellular signalling pathways involving APP and/or Tau may thus participate in synapse loss. Additionally, dysregulation of the core FA pathway could modulate APP and Tau metabolisms, leading to an exacerbation of synaptotoxicity through Aβ overproduction and Tau-modulated excitotoxicity. Finally, Aβ availability at the synapses is dependent on its clearance through the blood brain barrier and/or by microglial cells

When placing these new genetic and biological data into the broader context of known AD pathogenic mechanisms, several general conclusions seem to emerge, allowing us to propose an interpretation linking the main information resulting from this new genetic landscape into a global schema (Fig. 4):

1. The focus is shifted towards the synapse as the subcellular compartment where an important and early part of the pathogenesis takes place.
2. A genetic-dependent dysregulation of synapse dynamics and downstream cellular signalling through the core FA pathway may participate in AD processes, independently of Aβ toxicity.
3. The normal cellular functions of APP and Tau also appear to play central roles in synaptic plasticity, and their dysfunction may thus participate in the AD pathology, independently of the known toxic properties of their aggregated forms.
4. Synaptic dysfunction may thus simply link APP and Tau in the same subcellular compartment and APP functions may be also dependent on the FA pathway.
5. Aβ toxicity may, however, participate in several of these harmful dysfunctions, for instance through integrin over-activation. Under pathophysiological conditions, these dysfunctions might be either induced by massive Aβ peptide overproduction or accentuated by insidious Aβ accumulation through degradation/clearance failure. In monogenic forms of AD, Aβ would still be responsible for the pathogenesis, whereas in sporadic forms, Aβ would be a somewhat important accelerator of the pathological process.
6. Finally, genetic-dependent microglia dysfunction may take place in this synapse-centred model at two distinct levels: through Aβ clearance failure and/or synapse pruning. Aβ clearance failure may of course increase Aβ toxicity in the vicinity of synapses. It is also suspected that perturbations in homeostatic microglia functions at synapses may lead to early synapse loss in an Aβ-dependent or potentially independent manner [52, 93].

**Fig. 5:**
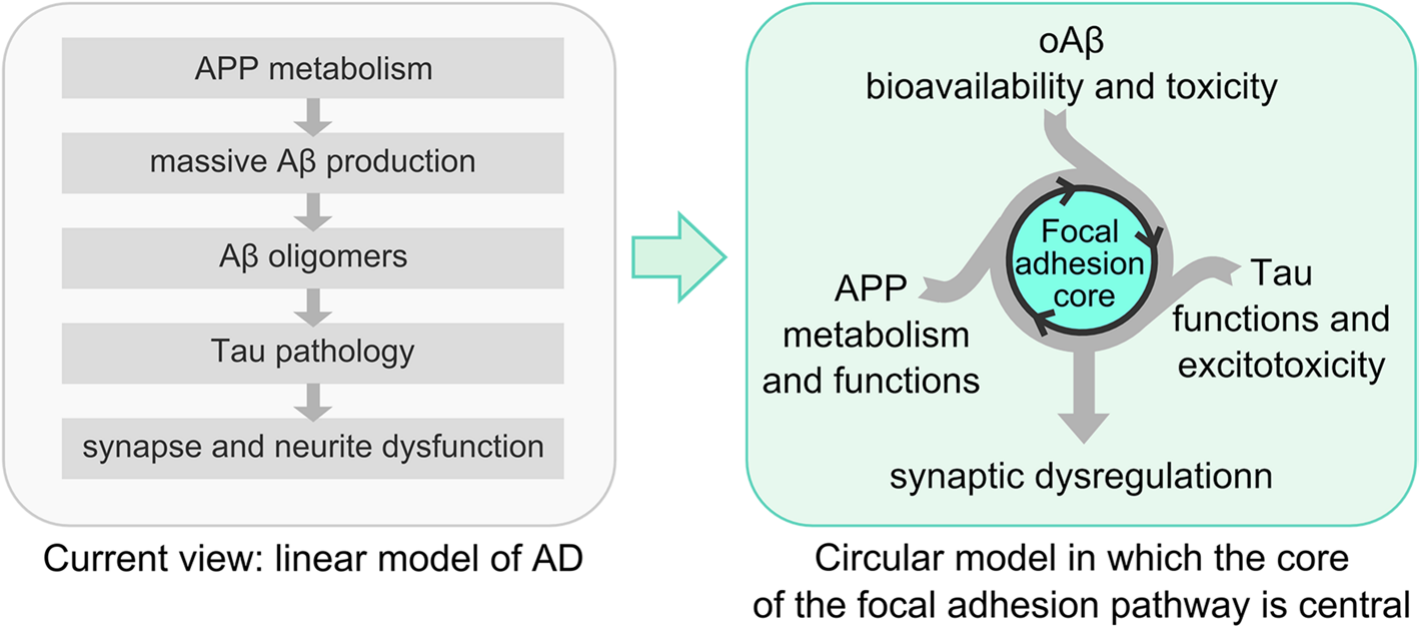
Reappraisal of the amyloid cascade hypothesis into a circular model. In this new model, dysfunction of the FA pathway at synapses could be the basis of a vicious cycle with multiple entry points linking AD hallmarks to synapse dysregulation, synapse loss, and subsequent cognitive decline

Within this background, dysregulation of at least one of these functions/processes (and/or of the crosstalk between them) may be sufficient to establish a vicious cycle leading to synaptic failure with multiple entry points (Fig. 5). This interpretation of the GWAS data does not exclude other hypotheses developed in the AD field:

1. Even if our interpretation implies the need to reappraise the amyloid cascade hypothesis from a linear model to an over-arching circular model, one could argue that our proposition is a reorganization of the various components of the amyloid cascade hypothesis and thus does not disqualify it. However, it is important to note that, since the model is no longer linear, Aβ peptides may be sufficient but not necessary to develop AD.
2. Similarly, the cellular phase hypothesis has recently been described to consist of “a long and complex phase consisting of feedback and feedforward responses of astrocytes, microglia and vasculature” [129]. These initially compensatory processes become subsequently irreversible and deleterious, leading to a progressive neurodegeneration. A synapse-centred hypothesis is not in contradiction with the latter since synapse homeostasis is strongly dependent on the interaction of multiple cell types in the brain. Genetic susceptibility may thus potentiate specific compensatory processes—particularly in the synapse—which become deleterious and lead to synapse loss. In addition, feedback and feedforward responses may be modified by genetic factors promoting or demoting synaptic failure. For instance, little is known about how AD genetic risk factors may modulate astrocyte functions in AD, and this aspect deserves to be studied, considering that a large part of the focal adhesion genes are also expressed in this cell type.
3. A synapse-centred hypothesis also does not preclude the spreading hypothesis [34, 66], which could be either a downstream pathological event or a potential trigger of Tau-related excitotoxicity and synaptic failure. One can argue that spreading of an AD-specific Tau strain [32] may be involved in one of the entry points of the vicious cycle, leading to synaptic failure. Notably, BIN1 has been reported to potentially modulate the spreading of Tau aggregates through their internalization via endocytosis and endosomal trafficking [14].

## Conclusions

Therapeutic approaches based on the classical amyloid cascade hypothesis have mostly failed, suggesting that our understanding of AD is far from being complete. The proportion of risk attributable to genetic susceptibility factors for AD has been estimated to be between 60 and 80% [30], which is exceptionally high for a common, multifactorial aging-related disease. The identification of this genetic variability and of the genes that carry it is, therefore, decisive for characterising the key pathophysiological elements in AD and for improving our understanding of its mechanisms and targets of interest. We thus hypothesized that exhaustive knowledge of the genetic determinants of AD and integration of their potential physiological and pathological functions would help better understand AD processes. This was made possible with the development of high-throughput genomic methodologies, e.g., GWAS and sequencing, potentially leading to an exhaustive overview of the AD genetic component in the medium-term. To date, more than 45 loci/genes have been characterised, and—keeping in mind the significant bias and tautological reasoning—numerous AD genetic risk factors appeared to support the amyloid cascade hypothesis through Aβ production or clearance. However, this strong genetic component also indicates—as suggested by other AD fields, e.g., imaging, biomarkers, disease models—that this hypothesis has to be reformulated to clearly describe AD and to take these recent advances into account. We thus propose an over-arching model that could help better define and target relevant pathophysiological processes. This approach led to the proposition that synaptic failure in AD may be one of the main pathophysiological events driven by genetics, and that the core of the FA pathway is a central node of this deleterious process. This model implies a vicious cycle that integrates the main actors of AD—APP and Tau—with the genetic risk factors identified for AD. This model also suggests that treatment options would depend on both the entry point into the vicious cycle and the interplay between different pathophysiological actors. For instance, therapies targeting Aβ peptides may not be effective if this entry point is relatively unimportant for a particular patient. It thus would be necessary to develop multiple therapies targeting different entry points. Once a range of treatment options become available, an individualized model of AD pathology would be feasible, such that polytherapies and personalised medicine approaches can be developed.

## Electronic supplementary material

Below is the link to the electronic supplementary material.

Supplementary material 1 (PDF 336 kb)
