# Supplementary material for: The new genetic landscape of Alzheimer’s disease: from amyloid cascade to genetically driven synaptic failure hypothesis?
Source: Acta Neuropathol. 2019 Apr 13;138(2):221–36. doi: 10.1007/s00401-019-02004-0 (PMC6660578; doi:10.1007/s00401-019-02004-0)
Supplement: Supplementary file 1 — Supplementary material 1 (PDF 336 kb) [file 401_2019_2004_MOESM1_ESM.pdf]

The New Genetic Landscape of Alzheimer's Disease: from Amyloid Cascade to Genetically-driven Synaptic Failure Hypothesis

Supplementary Material

Pierre Dourlen, Devrim Kilinc, Nicolas Malmanche, Julien Chapuis, Jean-Charles Lambert

Inserm, U1167, RID-AGE-Risk Factors and Molecular Determinants of Aging-Related Diseases, Lille, France; Institut Pasteur de Lille, Lille, France; University Lille, U1167-Excellence Laboratory LabEx DISTALZ, Lille, France.

Supplementary Table 1 Functional description of the genes located in the known AD risk loci. The name of the loci usually corresponds to the gene closest to the most significant genetic variant of the locus. Of note, it does not indicate that this gene is the AD functional gene of the locus. Information were manually curated from searches on PubMed (https://www.ncbi.nlm.nih.gov/pubmed/) using the name of the gene and Alzheimer and/or neuron as keywords.

Locus	Gene	General description	Relation to Aβ/APP	Relation to Tau	Other features
ABCA7 [19, 50, 95, 108, 138, 144, 240]	ABCA7	ATP binding cassette subfamily A member 7, member of the 'A' subfamily of ATPbinding cassette transporters initially characterized by their capacity to transport lipids across membranes [134]	- associated with plaque burden in AD brain [234]. Associated with Aβ deposition on in vivo imaging in human [13, 292]<br>- regulates APP processing resulting in an inhibition of Aβ production in vitro and in vivo in APP/PS1 mice, ABCA7 deletion facilitates the processing of APP to Aβ by increasing the levels of β-site APP cleaving enzyme 1 (BACE1) [35, 214, 219]<br>- stimulates phagocytosis in macrophages. ABCA7 loss results in 50% reduction of oAβ uptake by bone marrow-derived macrophages. ABCA7 loss doubles insoluble Aβ levels in J20 amyloidogenic mouse brain [110, 133]<br>- highly expressed in microglia. Its loss reduces microglia phagocytic clearance of amyloid-β [69]	- associated with neurofibrillary tangle pathology in AD brain [287]	- stimulates cellular cholesterol efflux to APOE discs [35]<br>- ABCA7 rs3764650 associated with cortical and hippocampal atrophy in MCI [203]<br>- genetic risk factor for posterior cortical atrophy (PCA), typically a rare variant of AD [221]<br>- its loss-of-function variants are enriched in patients with AD [78]<br>- Lysophosphatidylcholine is one of the major transport substrates for ABCA7 in the brain and this transport may be an important function of ABCA7 [254]
ABI3 [108, 237]	*ABI3*	ABI family member 3, adaptor protein with a homeobox homology domain, a proline rich region and a SH3 domain			- highly expressed in microglia cells, coexpressed with INPP5D, important role in actin cytoskeleton organization through participation in the WAVE2 complex, a complex that regulates multiple pathways leading to T cell activation [188, 222, 237]
AC099552.4 [25]	AC099552.4	long non coding RNA			

ACE [138]	ACE	Angiotensin I converting Enzyme, peptidase activity (reviewed in [127])	- ACE is an Aβ degrading enzyme [93, 99, 192, 249, 255]<br>- ACE expression in AD brain tissue is associated with Aβ load and AD severity. Exposing SH-SY-5Y neurons to oAβ1-42 increases ACE level and activity, suggesting Abeta may upregulate ACE in AD [179]<br>- CSF levels of the angiotensin-converting enzyme (ACE) are associated with Aβ levels [115] and LOAD risk [126]	-	- in CSF, its levels but not activity is reduced in AD [179]
	CYB561	cytochrome b561			
	TANC2	tetratricopeptide repeat, ankyrin repeat and coiled-coil containing 2, synaptic scaffold protein			- its overexpression in cultured neurons increases the density of dendritic spines and excitatory synapses [85]<br>- involved in the capture of KIF1A-driven dense core vesicle (DCV) at dendritic spines [244]
ADAM10 [108, 138]	ADAM10	the major α-secretase in the brain (reviewed in [213])	- component of the non-amyloidogenic pathway of APP metabolism (reviewed in [82]).<br>- over-expression of ADAM10 in mouse models can halt Aβ production and subsequent aggregation [199].<br>- two rare ADAM10 mutations segregating with disease in LOAD families increased Aβ plaque load in "Alzheimer-like" mice, with diminished α-secretase activity from the mutations likely the causal mechanism [130, 248]		-numerous and fundamental functions in the development of the embryonic brain and also in the homeostasis of adult neuronal networks. Mechanistically, ADAM10 controls these functions by utilizing unique postsynaptic substrates in the central nervous system, in particular synaptic cell adhesion molecules (reviewed in [213])<br>- sheds TREM2 to release soluble TREM2
ADAMTS1 [138]	ADAMTS1	a disintegrin and metalloproteinase with thrombospondin motifs 1			- manifold overexpressed in brain of AD patients [176]<br>- ADAMTS1 null female, but not male, exhibits a decline in synaptic protein levels [96]

ADAMTS4 [108]	ADAMTS4	a disintegrin and metalloproteinase with thrombospondin motifs 4	- generates N-truncated Aβ4-x species and marks oligodendrocytes as a source of amyloidogenic peptides in Alzheimer's disease [267]	- could modulate Tau phosphorylation by cleaving Reelin (reviewed in [80])	
	B4GALT3	beta-1,4-galactosyltransferase 3			
	PPOX	protoporphyrinogen oxidase			
AKAP9 [154]	AKAP9	A-kinase anchoring protein 9<br>AKAPs bind or tether protein kinase A (PKA) and other signaling molecules to relevant targets [275]		- AKAP9 mutations significantly increases pTau/Tau ratio in lymhoblastoid cell lines treated with phosphodiesterase-4 inhibitor rolipram, which activates protein kinase A [104]	
ALPK2 [108]	ALPK2	alpha kinase 2			
APH1B [108]	APH1B	aph-1 homolog B	- subunit of the gamma-secretase complex that cleaves APP [224]		
APOE [46]	APOE	apolipoprotein E<br>maintains the structure of specific lipoprotein particles and directs lipoproteins to specific cell surface receptors<br>3 alleles/isoforms, ε2, ε3, ε4, the first and the last being protective and deleterious respectively for Alzheimer's disease	- binding to Aβ<br>- role in Aβ production<br>- role in Aβ aggregation<br>- role in Aβ degradation and clearance (reviewed in [103])	- ApoE affects tau pathogenesis, neuroinflammation, and tau-mediated neurodegeneration independently of amyloid-β pathology [233]	- hypothesized to influence all the hallmarks of AD, from APP/Aβ to Tau aspects through lipid metabolism and neuroinflammation (reviewed in [243, 259])
BHMG1 / FBX046 [108]	BHMG1	basic helix-loop-helix and HMG-box containing 1			
	FBXO46	F-box protein 46, protein ubiquitin ligase			

BIN1 [108, 138, 144, 184, 225]	BIN1	bridging integrator 1, nucleocytoplasmic adaptor protein involved in endocytosis and membrane recycling, cytoskeleton regulation, DNA repair, cell cycle progression, and apoptosis [201]	- BIN1 depletion increases Aβ production through a decreased lysosomal BACE1 degradation [180]<br>- BIN1 depletion raises Aβ generation in axons by a defective BACE1 recycling to the membrane and increased BACE1 convergence with APP in early endosomes [260]<br>- BIN1 is found insoluble and accumulated in proximity to amyloid fibrils at the edges of amyloid deposits, suggesting a potential role for BIN1 in extracellular Aβ deposition in vivo [210]	-the functional risk allele (rs59335482) is associated with Tau loads in the brains of AD patients [37].<br>- loss of its Drosophila ortholog rescues Tau toxicity in Drosophila [37, 58]<br>- BIN1 SH3 domain interacts physically with Tau Proline-Rich domain and the interaction is regulated by Tau and BIN1 phosphorylation and an isoform-dependent BIN1 intramolecular folding [37, 163, 217, 239].<br>- lower BIN1 levels promote the propagation of Tau pathology by increasing aggregate internalization through regulation of endocytosis and endosomal trafficking [30]<br>- stabilizes Tau-induced actin bundles, loss of Drosophila BIN1 reduces tau-induced actin inclusions in Drosophila [60].	- Overexpression in mice results in neurodegeneration, loss of spine density, impaired LTP and behavioral deficits [52]<br>-interacts with integrin α3A in double hybrid screen and with the Focal Adhesion Kinase (FAK) [175, 273]<br>- physically interacts with intracellular form of CLU [299]
CASS4 [108, 138, 144]	CASS4	Cas scaffold protein family member 4, member of the CASS Scaffolding protein localized at focal adhesions, regulates cell spreading and motility [238]		- Its Drosophila ortholog modifies human Tau toxicity in Drosophila [58]<br>- associated with CSF Tau biomarker in AD patient [204]	-physical interaction between the CASS and FAK family of protein [16]
	CSTF1	cleavage stimulation factor (CSTF) subunit 1,<br>CSTF is involved in the polyadenylation and 3'end cleavage of pre-mRNAs			
	FAM209A	family with sequence similarity 209 member A			
	FAM209B	family with sequence similarity 209 member B			
	GCNT7	glucosaminyl (N-acetyl) transferase family member 7			

CASS4 (cont'd)	*RTFDC1*	replication termination factor 2 domain containing 1			
CD2AP [95, 108, 138, 144, 184]	CD2AP	CD2 associated protein, scaffolding protein involved in the regulation of membrane receptor endocytosis and signaling, actin cytoskeleton organization, endosomal vesicular trafficking, cell adhesion and cytokinesis [47, 61, 83, 116, 137, 157, 291]	-its loss-of-function raises Aβ generation in primary neuronal culture dendrite by increasing APP and its convergence with BACE1 in early endosomes but results in decreased cell membrane APP, decreased Aβ release and a lower Aβ42/Aβ40 ratio in N2a-APP695 cells. Complete loss of CD2AP results in a lower Aβ42/Aβ40 ratio in PS1APP mouse brain lysate but loss of one copy of CD2AP does not modify Abeta deposition or accumulation in these mice [152, 260]	- its Drosophila ortholog modifies human Tau toxicity in Drosophila [235]<br>- associated with CSF Tau biomarker in AD patient [204]	- decreased expression in peripheral blood lymphocytes from chinese sporadic AD patients [251]<br>- physically interacts with p130CAS (CASS1), colocalizes with F-actin and p130Cas to membrane ruffles and leading edges of cells in vitro, regulates actin cytoskeleton [135]<br>- coordinator of Neurotrophin Signaling-Mediated Axon Arbor Plasticity [91]<br>- regulates the signaling of the Tyrosine kinase Ret receptor in neurons [258]<br>- CD2AP-deficient mice have reduced blood-brain barrier integrity [44]<br>- recruited by RIN3 to RAB5a-positive early endosomes [211]
	GPR111	adhesion G protein-coupled receptor F2			
	GPR115	adhesion G protein-coupled receptor F4			

CD33 [95, 108, 184]	*CD33*	transmembrane sialic acid-binding immunoglobulin-like lectin that regulates innate immunity	- its expression is increased in microglial cells in AD brain and inhibits microglial uptake of Aβ, which correlates with increased Aβ42 levels and plaque burden in AD patient brain [27, 77]<br>- the AD-risk allele increases the full-length CD33M isoform containing the Exon2, which inhibit Aβ uptake [162, 202]. The protective CD33m isoform is localized in peroxisome and may be protective because it does not localize to the cell membrane and neither interact directly with amyloid plaques nor engage in cell-surface signaling [236]		
CELF1 / SPI1 [138, 144]	CELF1	CUGBP Elav-like family member 1, role in RNA processing (splicing and mRNA stability mainly), role in myotonic dystrophy (reviewed in [70])		- its Drosophila ortholog, Aret, modifies Tau toxicity in Drosophila [22, 235]	
	ACP2	acid phosphatase 2, lysosomal. lysosomal membrane member of the histidine acid phosphatase family, which hydrolyze orthophosphoric monoesters to alcohol and phosphate			- *Acp2* mutant mice exhibit ataxia due to degeneration of cerebellum neurons exhibiting lysosomal storage bodies [165]
	AGBL2	ATP/GTP binding protein like 2			
	C1QTNF4	C1q and TNF related 4			
	DDB2	damage specific DNA binding protein 2,<br>Protein that is necessary for the repair of ultraviolet light-damaged DNA	- oxidative stress generates a differential and specific DNA damage response involving overexpression of DDB2 in the presence of Aβ [67]		
	FAM180B	family with sequence similarity 180 member B			

CELF1 / SPI1 (cont'd)	*FNBP4*	formin binding protein 4,<br>protein containing two tryptophan-rich WW domains that binds the proline-rich formin homology 1 domains of formin family proteins, suggesting a role in the regulation of cytoskeletal dynamics during cell division and migration			
	KBTBD4	kelch repeat and BTB domain containing 4			
	MADD	MAP kinase activating death domain,<br>death domain-containing adaptor protein that interacts with the death domain of TNF-alpha receptor 1 to activate mitogen-activated protein kinase (MAPK) and regulate apoptosis	- change in MADD splice variants upon Aβ treatment, which could be protective [181]	- loss of its Drosophila ortholog enhances Tau toxicity in Drosophila [58]	- reduced expression and protein levels in the hippocampus of AD patients. MADD antisense treatment of cultured rat hippocampal neurons promoted neuronal cell death, suggesting a protective role of MADD in AD [265].
	MIR4487	microRNA 4487			
	MTCH2	mitochondrial carrier 2,<br>member of the SLC25 family of nuclear-encoded transporters that are localized in the inner mitochondrial membrane			- loss of forebrain MTCH2 in mice decreases mitochondria motility and calcium handling and impairs hippocampal-dependent cognitive functions [6, 212]
	MYBPC3	myosin binding protein C, cardiac.<br>cardiac isoform of myosin-binding protein C			
	NDUFS3	NADH:ubiquinone oxidoreductase core subunit S3,<br>one of the iron-sulfur protein (IP) components of mitochondrial NADH:ubiquinone oxidoreductase (complex I)	-RNA interference knockdown of the C. elegans ortholog of NDUFS3 is associated with Aβ toxicity [183]		

CELF1 / SPI1 (cont'd)	*NR1H3*	nuclear receptor subfamily 1 group H member 3. Also known as liver X receptor α (LXRA), member of the NR1 subfamily of the nuclear receptor superfamily, which are key regulators of macrophage function. Role in cholesterol metabolism and inflammation (reviewed in [31])	- genetic loss of either Lxrα or Lxrβ in APP/PS1 mice results in increased amyloid plaque load. Ligand activation of LXRs attenuates the inflammatory response of primary mixed glial cultures to fibrillar Aβ (fAβ) in a receptor-dependent manner and LXRs promote the capacity of microglia to maintain fAβ-stimulated phagocytosis in the setting of inflammation [288]		- involvment in cholesterol efflux in astrocytes [1]
	NUP160	Nucleoporin 160, one of the proteins that make up the 120-MD nuclear pore complex, which mediates nucleoplasmic transport			- the protective AD-associated rs9909-C allele in the 3'-UTR of NUP160 abolishes a miR target site and increases NUP160 expression, suggesting that increased expression of NUP160 might protect against the disease [53]
	PACSIN3	protein kinase C and casein kinase substrate in neurons 3, member of the PACSIN family of proteins involved in synaptic vesicular membrane trafficking and endocytosis [51]			
	PSMC3	proteasome 26S subunit, ATPase 3			
	PTPMT1	protein tyrosine phosphatase, mitochondrial 1			
	RAPSN	receptor associated protein of the synapse, also known as RAPSYN, critical role in clustering and anchoring nicotinic acetylcholine receptors at neuromuscular synaptic sites by linking the receptors to the underlying postsynaptic cytoskeleton, possibly by direct association with actin or spectrin.			
	SLC39A13	solute carrier family 39 member 13 member of the LIV-1 subfamily of the ZIP transporter family			

CELF1 / SPI1 (cont'd)	*SPI1*	Spi-1 proto-oncogene, ETS-domain transcription factor that activates gene expression during myeloid and B-lymphoid cell development	- the minor allele of rs1057233 (G) shows association with delayed AD onset and lowers expression of SPI1 in monocytes and macrophages. SPI1 encodes PU.1, a transcription factor critical for myeloid cell development and function. Altered PU.1 levels affects the expression of mouse orthologs of many AD risk genes and the phagocytic activity of mouse microglial cells [100]
	LOC101928943		

CLU [90, 108, 138, 143, 144, 184]	CLU	Clusterin, multifunctional apolipoprotein (J) involved in lipid metabolism, inflammation, apoptosis, cell cycle (reviewed in [190, 272])	- increased expression and levels in AD [23, 171]<br>- influences the regional distribution of Aβ [178]<br>- Aβ increases intracellular clusterin and decreases clusterin protein secretion [128]<br>- sequesters oAβ1–40 [185]<br>- complex effect of CLU on extracellullar Aβ aggregation depending on APOE, CLU:Aβ ratio and mode of aggregation – nucleation or elongation [17, 54, 55, 193, 284]:<br>- suppresses the toxicity of Aβ42 oligomers after they are formed in vivo [34]<br>- mediates the clearance of Aβ from the brain by binding to LRP2/megalin receptor at the level of the blood-brain and blood-cerebrospinal barrier [18, 84, 302]. Loss of clusterin shifts amyloid deposition to the cerebrovasculature via disruption of perivascular drainage pahtways [274].<br>- CLU regulates Aβ toxicity via Dickkopf-1-driven induction of the wnt-PCP-JNK pathway [128]	- its rs11136000 SNP is associated with Tau CSF levels modification in AD patients [299]<br>- its intracellular form physically interacts with Tau and BIN1 [299]<br>- its intracellular form is upregulated in the brain of Tau overexpressing Tg4510 mice [299]	- inhibition of the complement by binding to C5b-7 component [42]<br>- its non-synonymous mutation reduces the secretion of its protein [24]<br>- differentially expressed in the microglia of the 5xFAD mouse model [205]
	SCARA3	scavenger receptor class A member 3, express a macrophage scavenger receptor-like protein that would protect cells from oxidative stress			

CNTNAP2 [108]	CNTNAP2	contactin associated protein like 2, encodes a member of the neurexin family which functions in the vertebrate nervous system as cell adhesion molecules and receptors		- this protein is localized at the juxtaparanodes of myelinated axons, and mediates interactions between neurons and glia during nervous system development and is also involved in localization of potassium channels within differentiating axons.<br>- this gene has been implicated in multiple neurodevelopmental disorders
CR1 [108, 138, 143, 144, 184]	CR1	complement C3b/C4b receptor 1 (Knops blood group),<br>member of the receptors of complement activation (RCA) family [301]	- expressed on erythrocyte, clears Aβ from the blood by binding to circulating Aβ-C3b-complement component complexes [29, 208]<br>- association of CR1 risk allele and amyloid plaque burden/Amyloid deposition by in vivo brain imaging [41, 300]<br>- activation of microglial CR1 is detrimental to neurons and blockade of CR1 decreases the capacity of microglia to phagocytose Aβ1-42 [48]	- decrease expression of the long CR1 isoform, decrease CR1 density per erythrocyte and increased blood soluble CR1 in AD patients [159]
ECHDC3 [57, 108, 119, 138, 153]	ECHDC3	enoyl-CoA hydratase domain containing 3		- increased expression in AD brains compared to control [57]
	USP6NL	ubiquitin-specific peptidase 6 N-terminal like		- USP6NL is a Rab5 GTPase-activating protein, which regulate the endocytosis and internalization of EGFR [146, 167]<br>- USP6NL is also an effector of Rab5 that regulates the actin cytoskeleton [145]

EPHA1 [95, 108, 138, 144, 184]	EPHA1	Founding member of the Eph family of tyrosine kinase receptor [94]		- Drosophila ortholog of Eph receptor modifies human Tau toxicity in Drosophila [59]	- interacts with Integrin-Like Kinase and regulates cell morphology and motility through the ILK-RhoA-ROCK pathway [282]<br>- role of ephrin/EphR in synapse development and plasticity [140]<br>- altered distribution of its paralog EphA4 with neuritic plaques in AD [209]<br>- EphA4 receptor regulates dendritic spine remodeling by affecting β1-integrin signaling pathways [26]<br>- synaptic role of EphA4 in Aβ toxicity [68]<br>- EphA4 activation of c-Abl mediates synaptic loss and LTP blockade caused by Aβ oligomers [264]<br>- EphA4 cleaved by γ-secretase, γ-secretase-cleaved EphA4 intracellular domain regulates dendritic spine formation [105, 169]
	CASP2	caspase 2,<br>member of the cysteine-aspartic acid protease (caspase) family involved in apoptosis	- activated upon Aβ treatment [3, 166, 257]<br>- required for dendritic spine and behavioural alterations in J20 APP transgenic mice, as a critical mediator in the activation of the RhoA/ROCK-II signalling pathway, in the presence of Aβ [200]	- caspase-2 cleavage of tau at Asp314 impairs cognitive and synaptic function in animal and cellular models of tauopathies by promoting the missorting of tau to dendritic spines [293]	
	CLCN1	chloride voltage-gated channel 1, involved in the regulation of the electric excitability of the skeletal muscle membrane			- unexpected expression in human and mouse brain. Some CLCN1 variants are associated with epilepsy [38]
	EPHA1-AS1	EPHA1 antisense RNA 1			
	FAM131B	family with sequence similarity 131 member B			
	LOC100507507	uncharacterized LOC100507507			

EPHA1 (cont’d)	*TAS2R41*	taste 2 receptor member 41,<br>member of the bitter taste receptor family which belong to the G protein-coupled receptor superfamily and are predominantly expressed in taste receptor cells of the tongue and palate epithelia.			
	TAS2R60	taste 2 receptor member 60,<br>member of the bitter taste receptor family which belong to the G protein-coupled receptor superfamily and are predominantly expressed in taste receptor cells of the tongue and palate epithelia			
	ZYX	Zyxin,<br>zinc-binding phosphoprotein that concentrates at focal adhesions and along the actin cytoskeleton, may function as a messenger in the signal transduction pathway that mediates adhesion-stimulated changes in gene expression and may modulate the cytoskeletal organization of actin bundles			- its C. elegans ortholog regulates synapse maintenance [156]
	MIR6892	microRNA 6892			
FERMT2 [138, 144]	FERMT2	fermitin family member 2,<br>focal adhesion protein involved in integrin activation [141]	- increases Aβ peptide production by raising levels of mature APP at the cell surface and facilitating its recycling [36]<br>- associated with brain amyloidosis on in vivo brain imaging [13]	- its Drosophila ortholog modifies human Tau toxicity in Drosophila [235]	
	ERO1A	endoplasmic reticulum oxidoreductase 1 alpha			
	GNPNAT1	glucosamine-phosphate N-acetyltransferase 1			
	GPR137C	G protein-coupled receptor 137C			
	PSMC6	proteasome 26S subunit, ATPase 6			

FERMT2 (cont'd)	*STYX*	serine/threonine/tyrosine interacting protein,<br>it is a pseudophosphatase			
FRMD4A [142]	*FRMD4A*	FERM domain containing 4A,<br>FERM domain-containing protein that regulates epithelial cell polarity		- regulates Tau secretion in HEK293 cells and mature cortical neurones [283]	- a homozygous mutation in FRMD4A results in a syndrome of congenital microcephaly, intellectual disability and dysmorphism [65]
KAT8 [108]	KAT8	lysine acetyltransferase 8,<br>member of the MYST histone acetylase protein family			
	BCKDK	branched chain ketoacid dehydrogenase kinase,<br>regulator of the valine, leucine, and isoleucine catabolic pathways			- mutations in the gene BCKDK cause autism, epilepsy, microcephaly, neurobehavioral deficits and intellectual disability [71, 189]
HESX1 / IL17RD / APPL1 [108]	*HESX1*	HESX homeobox 1,<br>transcriptional repressor in the developing forebrain and pituitary gland			
	IL17RD	interleukin 17 receptor D			

HESX1 / IL17RD / APPL1 (cont'd)	*APPL1*	adaptor protein, phosphotyrosine interacting with PH domain and leucine zipper 1,	- After being recruited by the β-cleaved carboxy-terminal fragment of APP, the rab5 effector APPL1 mediates rab5 overactivation in Down syndrome and AD leading to pathologically accelerated endocytosis, endosome swelling and selectively impaired axonal transport of rab5 endosomes [132]		- in AD hippocampus and not in control, APPL1 accumulates perisomatically as granules around neurons and co-localizes with glutamate receptor 2 and ubiquitin, suggesting the possible involvement of APPL1 in the synaptic modifications in AD [195]<br>- an APPL1/Akt signaling complex regulates dendritic spine and synapse formation in hippocampal neurons [160]<br>- adaptor protein APPL1 couples synaptic NMDA receptor with neuronal prosurvival phosphatidylinositol 3-kinase/Akt pathway [268]<br>- APPL1 gates long-term potentiation through its plekstrin homology domain [63]
HLA [108, 138, 144]	*HLA-DQA1*<br>*HLA-DQA2*<br>*HLA-DQB1*<br>*HLA-DRA*<br>*HLA-DRB1*<br>*HLA-DRB5*	member of the Major Histocompatibility Complex Class II (MHCII)			-highly expressed on reactive microglia (reviewed in [266, 290])<br>- increased HLA-DR positive microglia in AD [170, 279]<br>- increased expression in monocytes and neutrophils in AD [64]<br>- reactive microglia in patients with senile dementia of the Alzheimer type are positive for the histocompatibility glycoprotein HLA-DR [172]
	LOC100294145				
	MIR3135B				
HS3ST1 [57, 108]	HS3ST1	heparan sulfate-glucosamine 3-sulfotransferase 1,<br>member of the heparan sulfate biosynthetic enzyme family			- HS3ST1 transcript expression is decreased in AD brains [57]

IGHG3 [25]	IGHG3	immunoglobulin heavy constant gamma 3,<br>member of the IgG family	- some IgG antibodies present in human plasma recognize conformational epitopes present on Aβ fibrils and oligomers [191]. The anti-amyloidogenic activity is a general property of free human Ig gamma heavy chains. The F1 heavy chain prevents in vitro fibril growth and reduces in vivo soluble Aβ oligomer-induced impairment of rodent hippocampal long term potentiation [2]		
INPP5D [108, 138, 144]	INPP5D	member of the inositol polyphosphate-5-phosphatase (INPP5) family		- associated with CSF Tau biomarker [204]	-role in immune response [161, 290]<br>- increased mRNA expression in the early stage which decreases with cognitive decline in Japanese AD subjects [286]
	NEU2	neuraminidase 2,<br>glycohydrolytic enzyme which removes sialic acid residues from glycoproteins and glycolipids			- neuramidases regulate many aspect of brain physiology at the level of the cell surface carbohydrates of the central nervous system tissues [76]<br>- NEU1 regulate APP metabolism through desialylation [12]
	NGEF	neuronal guanine nucleotide exchange factor,<br>member of the Dbl family of proteins, which function as guanine nucleotide exchange factors for the Rho-type GTPases			- NGEF links Eph receptors to the actin cytoskeleton and are involved in axon gowth cone dynamic [226]
IQCK [138]	IQCK	IQ motif containing K,<br>The IQ motif serves as a binding site for different EF-hand proteins such as calmodulin			
	KNOP1	lysine rich nucleolar protein 1			

IQCK (cont'd)	C16orf62	VPS35 endosomal protein sorting factor like,<br>component of the retriever complex, which recycles endocytosed membrane protein back to the cell membrane [173]			
MAPT [118]	MAPT	microtubule associated protein tau Composing one of AD hallmarks	too huge to be reviewed here	too huge to be reviewed here	too huge to be reviewed here
MS4A [95, 108, 138, 144, 184]	*MS4A2*<br>*MS4A3*<br>*MS4A4A*<br>*MS4A4E*<br>*MS4A6A*<br>*MS4A6E*<br>*MS4A7*	Members of a family of membrane proteins with four transmembrane domains. Role in calcium signaling and immune function (reviewed in [123, 158])	- elevated MS4A6A expression levels associated with Braak plaque score [124]	- expression of MS4A6A significantly increases in relation to increasing AD-related neurofibrillary pathology [168]	- MS4A4A LOAD risky allele associates with higher brain expression [5]
	OOSP2	oocyte secreted protein 2			
OARD1 [138]	*OARD1*	O-acyl-ADP-ribose deacylase 1, hydrolyzes mono-ADP-ribosylation and interacts with poly-ADP-ribose chains post-translational modifications			-homozygous mutation of the OARD1 gene in patients is associated with severe neurodegeneration [227]
PFDN1 / HBEGF [119, 153]	*PFDN1*	prefoldin subunit 1,<br>one of six subunits of prefoldin, a molecular chaperone complex that facilitates posttranslational folding of actins and other cytoskeletal proteins			-Pfdn1-deficient mice displayed phenotypes characteristic of defects in cytoskeletal function, including manifestations of ciliary dyskinesia, neuronal loss, and defects in B and T cell development and function [32]
	HBEGF	heparin binding EGF like growth factor			-HBEGF is an abundant neurotophic molecule of the brain regulating many higher brain functions [197]

PICALM [90, 108, 138, 144, 184]	PICALM	phosphatidylinositol binding clathrin assembly protein,<br>protein involved in clathrin mediated endocytosis (reviewed in [281])	- modifies Aβ toxicity in relationship with endocytosis in yeast [256]<br>- regulates APP endocytosis, subsequent APP metabolism and Aβ production [252, 280]<br>- binds LC3, suggestive role in the trafficking APP-CTF from the endocytic pathway to the autophagic degradation pathway and in Aβ clearance [253]<br>- regulates γ-secretase endocytosis and subsequent Aβ42/total Aβ ratio [120, 121]<br>- regulates Aβ blood-brain-barrier transcytosis and clearance [294]	- regulates autophagy, Tau clearance and Tau toxicity [182]<br>- co-localizes and coimmunoprecipitates with neurofibrillary tangles in human brains [10]<br>- its levels correlates with Tau pathology and autophagy impairments in human brains [11]	- strongly expressed in microglia [10]<br>- regulates the endocytosis of synaptic vesicle proteins [89, 177]<br>- regulates the cell surface level of the AMPA receptor subunit GluR2 [88]
	CCDC83	coiled-coil domain containing 83			
PLCG2 [237]	PLCG2	phospholipase C gamma 2, transmembrane signaling enzyme converting PIP2 into IP3 and DAG second messengers			-highly expressed in microglia cells and limited expression in neurons, oligodendrocytes, astrocytes, and endothelial cells [289]

PTK2B [138, 144]	PTK2B	protein tyrosine kinase 2 beta, encode the Pyk2 protein, member of the Focal Adhesion Kinase (FAK) family of protein tyrosine kinase	- mediates Aβ neurotoxicity downstream of integrins [276]<br>- its Aβo-induced phosphorylation is inhibited by Fyn inhibition [125]<br>- transduces signal downstream of Aβo-PrPC-mGluR5 complexes with deleterious effects on synaptic transmission and maintenance [81, 216]<br>- Hippocampal slices lacking Pyk2 are protected from AD-related Aβ oligomer suppression of synaptic plasticity. In APPswe/PSENΔE9 mice, deletion of Pyk2 rescues synaptic loss and learning/memory deficits [215]. Upon oAβ treatment, brain Pyk2 interacts with the RhoGAP protein Graf1 to alter dendritic spine stability via RhoA GTPase [148].<br>- less active in 5XFAD mice. Loss of Pyk2 in 5XFAD x Pyk2-/- double mutant mice has no effect but lentivirus-mediated Pyk2 overexpression improves synaptic markers and behavioral performance suggesting that Pyk2 is not essential for the pathogenic effects of human amyloidogenic mutations in the 5XFAD mouse model. However Pyk2 could contribute to amyloid plaque formation [74].<br>- activated in microglial cells upon fibrillar Aβ treatment [45]	- its Drosophila ortholog modifies human Tau toxicity in Drosophila [59]<br>- abnormally accumulates in neuronal somata concurrently with early markers of Tau pathology in brains of the Thy-Tau22 mouse model and AD patients [59]	- PTK2B is activated by neuronal depolarization, Ca2+ and stressful conditions [75]<br>- Role in neurite outgrowth [106]<br>- Role in LTP and LTD [97, 102]. On hippocampal slices, Pyk2 is not required for basal synaptic transmission or long term potentiation but participates in long term depression [215]<br>- Deficiency in mice have alterations in NMDA, PSD-95 and spines structures. Low level of PTK2B in huntington mouse model. Normalizing PTK2B levels rescues memory deficits, spines pathology and PSD-95 localization [72]<br>- Role in the survival of cerebellar granule neurons [242]<br>- Essential for astrocytes mobility following brain lesion [73]
	EPHX2	epoxide hydrolase 2,<br>The protein, found in both the cytosol and peroxisomes, binds to specific epoxides and converts them to the corresponding dihydrodiols.			-its products regulate synaptic plasticity [277]<br>- coding mutation in EPHX2 modifies the phenotype of familial hypercholoesterolemia [218]

PTK2B (cont’d)	*STMN4*	stathmin 4,<br>belongs to a family of proteins that regulates microtubule dynamics [49]	
	TRIM35	tripartite motif containing 35,<br>a RING-B-box-coiled-coil protein with apoptotic function	
	CHRNA2	cholinergic receptor nicotinic alpha 2 subunit,<br>subunit of the muscle and neuronal nicotinic acetylcholine receptor	- Cholinergic transmission is strongly involved in Alzheimer with a major focus on CHRN7 (reviewed in[230])
	MIR6842	microRNA 6842	
SCIMP / RABEP1 [108, 153]	SCIMP	SLP adaptor and CSK interacting membrane protein,<br>transmembrane adaptor protein that is expressed in antigen-presenting cells and is localized in the immunologic synapse	
	RABEP1	rabaptin, RAB GTPase binding effector protein 1,	-role in endocytosis in neurons as an effector of Rab5 [186]
SLC24A4 / RIN3 [108, 138, 144]	SLC24A4	solute carrier family 24 member 4,<br>potassium-dependent sodium-calcium exchanger expressed in the brain [150]	- role in Ca2+ signaling in neurons controling feeding and satiety [151]<br>- necessary for rapid response termination and proper adaptation of vertebrate olfactory sensory neurons [241]
	RIN3	Ras and Rab interactor 3,<br>guanine nucleotide exchange factor for RAB5 and RAB31	- RIN3 recruits CD2AP to RAB5a-positive early endosomes [211]

SORL1 [20, 108, 138, 144, 187, 207, 263]	SORL1	sortilin related receptor 1, transmembrane protein, member of the sortilin family of receptors [271]	-sorts APP into the retromer recycling pathway at the expense of the late endosomal pathway where APP undergoes β- and γ-secretase cleavage to generate Aβ. Loss of SORL1 results in increased Aβ levels. SORL1 interaction and sorting of APP is dependent on APP dimerization [9, 62, 194, 207]<br>- modulates EphA4, attenuates synaptotoxic EphA4 activation and cognitive impairment associated with Aβ-induced neurodegeneration in AD [101]		
SPPL2A [153]	SPPL2A	signal peptide peptidase like 2A, member of the GXGD family of aspartic proteases, homologue of the presenilins, located in late endosome and lysosome compartments			-Various roles in immune cells, protease of TMEM106b, a genetic risk factor for the development of frontotemporal lobar degeneration [174]
TM2D3 [107]	TM2D3	TM2 domain containing 3, the encoded protein contains a structural module related to that of the seven transmembrane domain G protein-coupled receptor superfamily	- preferentially influences uptake of Aβ aggregates during phagocytosis [86]		- Mutation in the Drosophila TM2D3 homolog, almondex, causes a phenotype similar to loss of Notch/Presenilin signaling [107]<br>- By functional transcomplementation in Drosophila, the rare TM2D3 variant associated with LOAD, P155L, is a functionally damaging allele [107]

TREM2 [20, 79, 108, 114, 117, 138, 237]	TREM2	triggering receptor expressed on myeloid cells 2, cell surface receptor of the immunoglobulin superfamily expressed on microglial cells (reviewed in [232, 261])	- oAβ1-42 binds TREM2 on microglia and activates microglial response in a TREM2-dependent manner. The effect of the disease-associated mutations of TREM2 on its binding affinity to oAβ1-42 is debated [149, 297]<br>- its loss alters microglia phagocytosis, including phagocytosis of Aβ [111, 136]<br>- its TREM2 in amyloid mouse models (APPPS1-21, 5XFAD) results in defective microgliosis surrounding Aβ plaques with contradictory effects on the Aβ accumulation, whereas overexpression of TREM2 in the brain of APPswe/PS1dE9 mice ameliorated AD-related neuropathology [109, 111, 262, 269]<br>- in vitro, human stem cell-derived monocytes and transdifferentiated microglia-like cells reveal impaired amyloid plaque clearance upon heterozygous or homozygous loss of TREM2 [43]<br>- its deficiency reduces the efficacy of immunotherapeutic amyloid clearance [278]	-increased soluble TREM2 (sTREM2) CSF levels in AD patients that correlates with CSF Tau levels [198, 246]<br>-its loss exacerbates Tau pathology in P301S Tau mice [112]. Increased TREM2 ameliorates the pathological effects of activated microglia on GSK3-mediated neuronal Tau hyperphosphorylation via suppression of microglial inflammatory response [113]<br>- In contrast, glial expression of TREM2/TYROBP exacerbates Tau-mediated neurodegeneration in Drosophila [223]<br>- In transgenic mice expressing mutant human tau, TREM2 haploinsufficiency, but not complete loss of TREM2, increases Tau pathology. The effect on microglial injury and Tauopathy would depend on the partial or complete loss of TREM2 [220]	- Promotes Microglial Survival by Activating Wnt/β-Catenin Pathway [295]<br>- The AD-associated R47H TREM2 mutation have pleiotropic negative effects on microglia and myeloid cells that can be rescued by some TREM2-activating antibodies [39, 40]<br>- Structural analysis reveals that Arg47 plays a critical role in maintaining the structural features of the complementarity-determining region 2 (CDR2) loop and the putative positive ligand-interacting surface (PLIS), stabilizing conformations capable of ligand interaction [247]. However AD-associated TREM2 mutant would bind Aβ with equivalent affinity but show loss of function in terms of signaling and Aβ internalization [149]<br>- APOE is a ligand of TREM2 whose binding to TREM2 can be blocked by the high-affinity binding of oAβ to TREM2. APOE binding is reduced for R47H TREM2 [14, 15, 149]
	TREML2	triggering receptor expressed on myeloid cells like 2, structurally related to the TREM famlily but does not signal though DAP12/TYROBP (reviewed in [66])	- oligomeric amyloid-β treatment up-regulates TREML2 expression in primary microglia [296]		- has AD-associated functional variants independent of TREM2 ones [21].<br>- The modulation of TREM2 or TREML2 levels has opposing effect on the proliferation of primary microglia, TREM2 or TREML2 downregulation respectively decrease or increase microglia proliferation [296].

	TSPOAP1	TSPO associated protein 1,<br>TSPO (translocator protein) is a key factor in the flow of cholesterol into mitochondria to permit the initiation of steroid hormone synthesis			
TSPOAP1 [108, 119]	MIR142	microRNA 142,<br>abundantly expressed in hematopoietic cells with roles in inflammatory and immune responses (reviewed [228])		-upregulated in the hippocampi of rTg4510 Tau mice in the presymptomatic stage and onward. Similar to what is observed in Tau brains, overexpressing miR142 in wildtype cortical neurons augments mRNA levels of *Gfap* and *Csf1*, accompanied by a significant increase in microglia and reactive astrocyte numbers [229]	
	TSPOAP1-AS1	TSPOAP1 antisense RNA 1			
WWOX / MAF [138]	WWOX	WW domain containing oxidoreductase,<br>member of the short-chain dehydrogenases/reductases (SDR) protein family		- WWOX is decreased in the neurons of AD hippocampi. WWOX binds to Tau through its SDR domain. Knock-down of WWOX results in Tau hyperphosphorylation [250]	- WWOX is mutated in autosomal recessive cerebellar ataxia with epilepsy and mental retardation [164]
	MAF	MAF bZIP transcription factor,<br>DNA-binding, leucine zipper-containing transcription factor that acts as a homodimer or as a heterodimer			- MAF promotes functional differentiation and anti-inflammatory responses in myeloid cells [33]<br>- Negative regulation of MAF mediates p53 proinflammatory responses in microglia [245]<br>- Maf links Neuregulin1 signaling to cholesterol synthesis in myelinating Schwann cells [131]<br>- c-Maf is required for the development of dorsal horn laminae III/IV neurons and mechanoreceptive DRG axon projections [98]

	ZCWPW1	Contain a zf-CW domain involved in histone modification reading [92]	
	ACTL6B	actin like 6B,<br>member of a family of actin-related proteins (ARPs)	-subunit of neuron-specific chromatin remodeling complex [196]<br>- its loss in mice results in synaptic plasticity and cocaine-associated memory defects that can be rescued by BDNF [270]
	AGFG2	ArfGAP with FG repeats 2,<br>member of the HIV-1 Rev binding protein (HRB) family and plays a role in the Rev export pathway, which mediates the nucleocytoplasmic transfer of proteins and RNAs	
ZCWPW1 / NYAP1 [108, 138, 144]	*AP4M1*	adaptor related protein complex 4 subunit mu 1,<br>subunit of the heterotetrameric AP-4 complex, which is involved in the recognition and sorting of cargo proteins with tyrosine-based motifs from the trans-golgi network to the endosomal-lysosomal system	-its mutation and AP-4 deficiency results in many clinical neuronal symptoms such as intellectual disability or hereditary spastic paraplegia…
	AZGP1	alpha-2-glycoprotein 1, zinc-binding,<br>encode Zinc-α2-glycoprotein (ZAG), which is a major histocompatibility complex I molecule and a lipid-mobilizing factor.	
	C7orf43	chromosome 7 open reading frame 43	
	C7orf61	chromosome 7 open reading frame 61	
	CNPY4	canopy FGF signaling regulator 4,<br>regulates cell surface expression of Toll-like receptor 4	

ZCWPW1 / NYAP1 (cont'd)	*COPS6*	COP9 signalosome subunit 6, one of the eight subunits of COP9 signalosome, regulator in multiple signaling pathways, whose structure is similar to that of the 19S regulatory particle of 26S proteasome.	
	CYP3A43	cytochrome P450 family 3 subfamily A member 43, The cytochrome P450 proteins are monooxygenases which catalyze many reactions involved in drug metabolism and synthesis of cholesterol, steroids and other lipids	
	FBXO24	F-box protein 24, F-box protein of the Fbxs class, the F-box proteins constitute one of the four subunits of the SCF ubiquitin protein ligase complex	
	GAL3ST4	galactose-3-O-sulfotransferase 4, member of the galactose-3-O-sulfotransferase protein family	
	CASTOR3	CASTOR family member 3, also named GATS	- Lower expression in AD brain [122]
	GIGYF1	GRB10 interacting GYF protein 1, member of the gyf family of adaptor proteins	- its Drosophila ortholog regulates neuronal autophagy [129]<br>- regulates the Insulin signaling pathway [298]
	GJC3	gap junction protein gamma 3	-expressed in the myelinating glial cells [8]
	GNB2	G protein subunit beta 2, G proteins integrate signals between receptors and effector proteins	

ZCWPW1 / NYAP1 (cont'd)	*GPC2*	glypican 2,<br>Glypicans are a group of cell-surface glycoproteins in which heparan sulfate (HS) glycosaminoglycan chains are covalently linked to a protein core, it can function as coreceptors for multiple signaling molecules.	- involved in neuronal cell adhesion and neurite outgrowth [139]
	LAMTOR4	late endosomal/lysosomal adaptor, MAPK and MTOR activator 4,<br>subunit of the Rag-Ragulator complex, located on lysosome membrane and regulating MAPK and mTOR signaling pathways	- Lamtor4 is an essential regulator of microglia lysosomes for proper lysosome function and phagocytic flux in zebrafish microglia [231]
	LOC100128334	gap junction protein gamma 3 pseudogene	
	LRCH4	leucine rich repeats and calponin homology domain containing 4,<br>the encoded protein resembles a membrane receptor	- Lrch4 is a Toll-Like Receptor accessory protein, which regulates innate immune response [7]
	MBLAC1	metallo-beta-lactamase domain containing 1	- differentially expressed in AD brains [28]<br>- C.elegans MBLAC1 limits Dopaminergic (DA) neuron excitability, DA secretion, and DA-dependent behaviors through modulation of Glu signaling [87]
	MCM7	minichromosome maintenance complex component 7,<br>subunit of the MCM complex, key component of the pre-replication complex, involved in the formation of replication forks and possessing a DNA helicase activity	
	MEPCE	methylphosphate capping enzyme, possesses histone-binding and RNA methylation activities involved in the regulation of transcription	

ZCWPW1 / NYAP1 (cont'd)	*MIR106B*	microRNA 106b	- reduces ATG16L1 levels and autophagy In human cell lines (Lu C et al 2014)[155]
	MIR25	microRNA 25	
	MIR4658	microRNA 4658	
	MIR93	microRNA 93	- reduces ATG16L1 levels and autophagy In human cell lines [155]
	MOSPD3	motile sperm domain containing 3, encodes a multi-pass membrane protein with a major sperm protein (MSP) domain	
	NYAP1	neuronal tyrosine phosphorylated phosphoinositide-3-kinase adaptor 1	- NYAP family links PI3K to WAVE1 complex, which mediates remodelling of the actin cytoskeleton, and regulate neuronal morphogenesis [285]
	OR2AE1	olfactory receptor family 2 subfamily AE member 1	
	PCOLCE	procollagen C-endopeptidase enhancer,<br>encodes a glycoprotein which binds and drives the enzymatic cleavage of type I procollagen and heightens C-proteinase activity	
	PCOLCE-AS1	PCOLCE antisense RNA 1	
	PILRA	paired immunoglobin like type 2 receptor alpha,<br>encode a cell surface inhibitory receptor paired with PILRB that recognizes specific O-glycosylated proteins and is expressed on various innate immune cell types including microglia	- The common missense variant (G78R, rs1859788) of PILRA could be the causal allele for the locus. The G78R mutation reduces the binding of PILRA to its ligand such as complement component 4A. It could protects individuals from AD risk via reduced inhibitory signaling in microglia [206]

ZCWPW1 / NYAP1 (cont'd)	*PILRB*	paired immunoglobin-like type 2 receptor beta,<br>encode a cell surface activator receptor paired with PILRB that recognizes specific O-glycosylated proteins and is expressed on various innate immune cell types including microglia	- its levels is associated with AD status [122] and with LOAD GWAS index SNPs [4]
	PPP1R35	protein phosphatase 1 regulatory subunit 35,<br>a centrosomal protein critical for centriole elongation	
	PVRIG	PVR related immunoglobulin domain containing,<br>a member of poliovirus receptor-like proteins and a coinhibitory receptor for human T cells	
	SAP25	Sin3A associated protein 25,<br>SAP25 associates with the mSin3A-HDAC complex in vivo and represses transcription	
	SPDYE3	speedy/RINGO cell cycle regulator family member E3	
	STAG3	stromal antigen 3,<br>subunit of the cohesin complex which regulates the cohesion of sister chromatids during cell division	
	TAF6	TATA-box binding protein associated factor 6,<br>component of the transcription factor IID involved in basal transcription	
	TFR2	transferrin receptor 2,<br>This protein mediates cellular uptake of transferrin-bound iron	
	TRIM4	tripartite motif containing 4	

ZCWPW1 / NYAP1 (cont'd)	*TSC22D4*	TSC22 domain family member 4, member of the TSC22 domain family of leucine zipper transcriptional regulators		
	ZKSCAN1	zinc finger with KRAB and SCAN domains 1,<br>encodes a member of the Kruppel C2H2-type zinc-finger family of proteins that may function as a transcription factor regulating the expression of GABA type-A receptors in the brain		
	ZNF3	zinc finger protein 3		
	ZSCAN21	zinc finger and SCAN domain containing 21		-ZSCAN21 regulates α-synuclein transcription in neuronal cells and rare genetic variants in ZSCAN21 gene occur in patients with familial forms of Parkinson's Disease [56, 147]
ZNF655 [25]	ZNF655	zinc finger protein 655		

References

1. Abildayeva K, Jansen PJ, Hirsch-Reinshagen V, Bloks VW, Bakker AHF, Ramaekers FCS, et al. (2006) 24(*S*)-Hydroxycholesterol Participates in a Liver X Receptor-controlled Pathway in Astrocytes That Regulates Apolipoprotein E-mediated Cholesterol Efflux. J Biol Chem 281:12799–12808. doi: 10.1074/jbc.M601019200

2. Adekar SP, Klyubin I, Macy S, Rowan MJ, Solomon A, Dessain SK, et al. (2010) Inherent anti-amyloidogenic activity of human immunoglobulin gamma heavy chains. J Biol Chem 285:1066–74. doi: 10.1074/jbc.M109.044321

3. Allen JW, Eldadah BA, Huang X, Knoblach SM, Faden AI (2001) Multiple caspases are involved in ?-amyloid-induced neuronal apoptosis. J Neurosci Res 65:45–53. doi: 10.1002/jnr.1126

4. Allen M, Kachadoorian M, Carrasquillo MM, Karhade A, Manly L, Burgess JD, et al. (2015) Late-onset Alzheimer disease risk variants mark brain regulatory loci. Neurol Genet 1:e15. doi: 10.1212/NXG.0000000000000012

5. Allen M, Zou F, Chai HS, Younkin CS, Crook J, Shane Pankratz V, et al. (2012) Novel late-onset Alzheimer disease loci variants associate with brain gene expression. Neurology 79:221–228. doi: 10.1212/WNL.0b013e3182605801

6. Aloni E, Ruggiero A, Gross A, Segal M (2018) Learning Deficits in Adult Mitochondria Carrier Homolog 2 Forebrain Knockout Mouse. Neuroscience 394:156–163. doi: 10.1016/j.neuroscience.2018.10.035

7. Aloor JJ, Azzam KM, Guardiola JJ, Gowdy KM, Madenspacher JH, Gabor KA, et al. (2018) Leucine-Rich Repeats and Calponin Homology containing 4 regulates the innate immune response. J Biol Chem jbc.RA118.004300. doi: 10.1074/jbc.RA118.004300

8. Altevogt BM, Kleopa KA, Postma FR, Scherer SS, Paul DL (2002) Connexin29 is uniquely distributed within myelinating glial cells of the central and peripheral nervous systems. J Neurosci 22:6458–70

9. Andersen OM, Reiche J, Schmidt V, Gotthardt M, Spoelgen R, Behlke J, et al. (2005) Neuronal sorting protein-related receptor sorLA/LR11 regulates processing of the amyloid precursor protein. Proc Natl Acad Sci U S A 102:13461–6. doi: 10.1073/pnas.0503689102

10. Ando K, Brion JP, Stygelbout V, Suain V, Authelet M, Dedecker R, et al. (2013) Clathrin adaptor CALM/PICALM is associated with neurofibrillary tangles and is cleaved in Alzheimer's brains. Acta Neuropathol 125:861–878. doi: 10.1007/s00401-013-1111-z

11. Ando K, Tomimura K, Sazdovitch V, Suain V, Yilmaz Z, Authelet M, et al. (2016) Level of PICALM, a key component of clathrin-mediated endocytosis, is correlated with levels of phosphotau and autophagy-related proteins and is associated with tau inclusions in AD, PSP and Pick disease. Neurobiol Dis 94:32–43. doi: 10.1016/j.nbd.2016.05.017

12. Annunziata I, Patterson A, Helton D, Hu H, Moshiach S, Gomero E, et al. (2013) Lysosomal NEU1 deficiency affects amyloid precursor protein levels and amyloid-β secretion via deregulated lysosomal exocytosis. Nat Commun 4:2734. doi: 10.1038/ncomms3734

13. Apostolova LG, Risacher SL, Duran T, Stage EC, Goukasian N, West JD, et al. (2018) Associations of the Top 20 Alzheimer Disease Risk Variants With Brain Amyloidosis. JAMA Neurol 75:328. doi: 10.1001/jamaneurol.2017.4198

14. Atagi Y, Liu C-C, Painter MM, Chen X-F, Verbeeck C, Zheng H, et al. (2015) Apolipoprotein E Is a Ligand for Triggering Receptor Expressed on Myeloid Cells 2 (TREM2). J Biol Chem 290:26043–26050. doi: 10.1074/jbc.M115.679043

15. Bailey CC, Devaux LB, Farzan M (2015) The triggering receptor expressed on myeloid cells 2 binds apolipoprotein E. J Biol Chem 290:26033–26042. doi: 10.1074/jbc.M115.677286

16. Beck TN, Nicolas E, Kopp MC, Golemis EA (2014) Adaptors for disorders of the brain? The cancer signaling proteins NEDD9, CASS4, and PTK2B in Alzheimer's disease. Oncoscience 1:486–503. doi: 10.18632/oncoscience.64

17. Beeg M, Stravalaci M, Romeo M, Carrá AD, Cagnotto A, Rossi A, et al. (2016) Clusterin Binds to Aβ1-42 Oligomers with High Affinity and Interferes with Peptide Aggregation by Inhibiting Primary and Secondary Nucleation. J Biol Chem 291:6958–66. doi: 10.1074/jbc.M115.689539

18. Bell RD, Sagare AP, Friedman AE, Bedi GS, Holtzman DM, Deane R, et al. (2007) Transport Pathways for Clearance of Human Alzheimer's Amyloid β-Peptide and Apolipoproteins E and J in the Mouse Central Nervous System. J Cereb Blood Flow Metab 27:909–918. doi: 10.1038/sj.jcbfm.9600419

19. Bellenguez C, Charbonnier C, Grenier-Boley B, Quenez O, Le Guennec K, Nicolas G, et al. (2017) Contribution to Alzheimer's disease risk of rare variants in TREM2, SORL1, and ABCA7 in 1779 cases and 1273 controls. Neurobiol Aging 59:220.e1–220.e9. doi: 10.1016/j.neurobiolaging.2017.07.001

20. Bellenguez C, Charbonnier C, Grenier-Boley B, Quenez O, Le Guennec K, Nicolas G, et al. (2017) Contribution to Alzheimer's disease risk of rare variants in TREM2, SORL1, and ABCA7 in 1779 cases and 1273 controls. Neurobiol Aging 59:220.e1–220.e9. doi: 10.1016/j.neurobiolaging.2017.07.001

21. Benitez BA, Jin SC, Guerreiro R, Graham R, Lord J, Harold D, et al. (2014) Missense variant in TREML2 protects against Alzheimer's disease. Neurobiol Aging 35:1510.e19–1510.e26. doi: 10.1016/j.neurobiolaging.2013.12.010

22. Bernstein AI, Lin Y, Street RC, Lin L, Dai Q, Yu L, et al. (2016) 5-Hydroxymethylation-associated epigenetic modifiers of Alzheimer's disease modulate Tau-induced neurotoxicity. Hum Mol Genet 25:2437–2450. doi: 10.1093/hmg/ddw109

23. Bertrand P, Poirier J, Oda T, Finch CE, Pasinetti GM (1995) Association of apolipoprotein E genotype with brain levels of apolipoprotein E and apolipoprotein J (clusterin) in Alzheimer disease. Brain Res Mol Brain Res 33:174–8

24. Bettens K, Vermeulen S, Van Cauwenberghe C, Heeman B, Asselbergh B, Robberecht C, et al. (2015) Reduced secreted clusterin as a mechanism for Alzheimer-associated CLU mutations. Mol Neurodegener 10:30. doi: 10.1186/s13024-015-0024-9

25. Bis JC, Jian X, Kunkle BW, Chen Y, Hamilton-Nelson KL, Bush WS, et al. (2018) Whole exome sequencing study identifies novel rare and common Alzheimer's-Associated variants involved in immune response and transcriptional regulation. Mol Psychiatry. doi: 10.1038/s41380-018-0112-7

26. Bourgin C, Murai KK, Richter M, Pasquale EB (2007) The EphA4 receptor regulates dendritic spine remodeling by affecting beta1-integrin signaling pathways. J Cell Biol 178:1295–307. doi: 10.1083/jcb.200610139

27. Bradshaw EM, Chibnik LB, Keenan BT, Ottoboni L, Raj T, Tang A, et al. (2013) CD33 Alzheimer's disease locus: altered monocyte function and amyloid biology. Nat Neurosci 16:848–850. doi: 10.1038/nn.3435

28. Broce IJ, Tan CH, Fan CC, Jansen I, Savage JE, Witoelar A, et al. (2018) Dissecting the genetic relationship between cardiovascular risk factors and Alzheimer's disease. Acta Neuropathol. doi: 10.1007/s00401-018-1928-6

29. Brubaker WD, Crane A, Johansson JU, Yen K, Garfinkel K, Mastroeni D, et al. (2017) Peripheral complement interactions with amyloid β peptide: Erythrocyte clearance mechanisms. Alzheimer's Dement 13:1397–1409. doi: 10.1016/j.jalz.2017.03.010

30. Calafate S, Flavin W, Verstreken P, Moechars D (2016) Loss of Bin1 Promotes the Propagation of Tau Pathology. Cell Rep 17:931–940. doi: 10.1016/j.celrep.2016.09.063

31. Cao G, Bales KR, DeMattos RB, Paul SM (2007) Liver X receptor-mediated gene regulation and cholesterol homeostasis in brain: relevance to Alzheimer's disease therapeutics. Curr Alzheimer Res 4:179–84

32. Cao S, Carlesso G, Osipovich AB, Llanes J, Lin Q, Hoek KL, et al. (2008) Subunit 1 of the prefoldin chaperone complex is required for lymphocyte development and function. J Immunol 181:476–84

33. Cao S, Liu J, Song L, Ma X (2005) The protooncogene c-Maf is an essential transcription factor for IL-10 gene expression in macrophages. J Immunol 174:3484–92

34. Cascella R, Conti S, Tatini F, Evangelisti E, Scartabelli T, Casamenti F, et al. (2013) Extracellular chaperones prevent Aβ42-induced toxicity in rat brains. Biochim Biophys Acta - Mol Basis Dis 1832:1217–1226. doi: 10.1016/j.bbadis.2013.04.012

35. Chan SL, Kim WS, Kwok JB, Hill AF, Cappai R, Rye K-A, et al. (2008) ATP-binding cassette transporter A7 regulates processing of amyloid precursor protein in vitro. J Neurochem 106:793–804. doi: 10.1111/j.1471-4159.2008.05433.x

36. Chapuis J, Flaig A, Grenier-Boley B, Eysert F, Pottiez V, Deloison G, et al. (2017) Genome-wide, high-content siRNA screening identifies the Alzheimer's genetic risk factor FERMT2 as a major modulator of APP metabolism. Acta Neuropathol 133:955–966. doi: 10.1007/s00401-016-1652-z

37. Chapuis J, Hansmannel F, Gistelinck M, Mounier A, Van Cauwenberghe C, Kolen K V, et al. (2013) Increased expression of BIN1 mediates Alzheimer genetic risk by modulating tau pathology. Mol Psychiatry 18:1225–34. doi: 10.1038/mp.2013.1

38. Chen TT, Klassen TL, Goldman AM, Marini C, Guerrini R, Noebels JL (2013) Novel brain expression of ClC-1 chloride channels and enrichment of CLCN1 variants in epilepsy. Neurology 80:1078–1085. doi: 10.1212/WNL.0b013e31828868e7

39. Cheng Q, Danao J, Talreja S, Wen P, Yin J, Sun N, et al. (2018) TREM2-activating antibodies abrogate the negative pleiotropic effects of the Alzheimer's disease variant *Trem2* ^{R47H} on murine myeloid cell function. J Biol Chem 293:12620–12633. doi: 10.1074/jbc.RA118.001848

40. Cheng-Hathaway PJ, Reed-Geaghan EG, Jay TR, Casali BT, Bemiller SM, Puntambekar SS, et al. (2018) The Trem2 R47H variant confers loss-of-function-like phenotypes in Alzheimer's disease. Mol Neurodegener 13:29. doi: 10.1186/s13024-018-0262-8

41. Chibnik LB, Shulman JM, Leurgans SE, Schneider JA, Wilson RS, Tran D, et al. (2011) *CR1* is associated with amyloid plaque burden and age-related cognitive decline. Ann Neurol 69:560–569. doi: 10.1002/ana.22277

42. Choi-Miura NH, Sakamoto T, Tobe T, Nakano Y, Tomita M (1993) The role of HDL consisting of SP-40,40, apo A-I, and lipids in the formation of SMAC of complement. J Biochem 113:484–7

43. Claes C, Van Den Daele J, Boon R, Schouteden S, Colombo A, Monasor LS, et al. (2018) Human stem cell-derived monocytes and microglia-like cells reveal impaired amyloid plaque clearance upon heterozygous or homozygous loss of TREM2. Alzheimers Dement. doi: 10.1016/j.jalz.2018.09.006

44. Cochran JN, Rush T, Buckingham SC, Roberson ED (2015) The Alzheimer's disease risk factor CD2AP maintains blood-brain barrier integrity. Hum Mol Genet 24:6667–74. doi: 10.1093/hmg/ddv371

45. Combs CK, Johnson DE, Cannady SB, Lehman TM, Landreth GE (1999) Identification of microglial signal transduction pathways mediating a neurotoxic response to amyloidogenic fragments of beta-amyloid and prion proteins. J Neurosci 19:928–39

46. Corder EH, Saunders AM, Strittmatter WJ, Schmechel DE, Gaskell PC, Small GW, et al. (1993) Gene dose of apolipoprotein E type 4 allele and the risk of Alzheimer's disease in late onset families. Science 261:921–3

47. Cormont M, Metón I, Mari M, Monzo P, Keslair F, Gaskin C, et al. (2003) CD2AP/CMS regulates endosome morphology and traffic to the degradative pathway through its interaction with Rab4 and c-Cbl. Traffic 4:97–112

48. Crehan H, Hardy J, Pocock J (2013) Blockage of CR1 prevents activation of rodent microglia. Neurobiol Dis 54:139–149. doi: 10.1016/j.nbd.2013.02.003

49. Curmi PA, Gavet O, Charbaut E, Ozon S, Lachkar-Colmerauer S, Manceau V, et al. (1999) Stathmin and its phosphoprotein family: general properties, biochemical and functional interaction with tubulin. Cell Struct Funct 24:345–57

50. Cuyvers E, De Roeck A, Van den Bossche T, Van Cauwenberghe C, Bettens K, Vermeulen S, et al. (2015) Mutations in ABCA7 in a Belgian cohort of Alzheimer's disease patients: a targeted resequencing study. Lancet Neurol 14:814–822. doi: 10.1016/S1474-4422(15)00133-7

51. D'hoedt D, Owsianik G, Prenen J, Cuajungco MP, Grimm C, Heller S, et al. (2008) Stimulus-specific Modulation of the Cation Channel TRPV4 by PACSIN 3. J Biol Chem 283:6272–6280. doi: 10.1074/jbc.M706386200

52. Daudin R, Marechal D, Wang Q, Abe Y, Bourg N, Sartori M, et al. (2018) BIN1 genetic risk factor for Alzheimer is sufficient to induce early structural tract alterations in entorhinal cortex-dentate gyrus pathway and related hippocampal multi-scale impairments. bioRxiv. doi: https://doi.org/10.1101/437228

53. Delay C, Grenier-Boley B, Amouyel P, Dumont J, Lambert J-C (2016) miRNA-dependent target regulation: functional characterization of single-nucleotide polymorphisms identified in genome-wide association studies of Alzheimer's disease. Alzheimers Res Ther 8:20. doi: 10.1186/s13195-016-0186-x

54. DeMattos RB, Cirrito JR, Parsadanian M, May PC, O'Dell MA, Taylor JW, et al. (2004) ApoE and clusterin cooperatively suppress Abeta levels and deposition: evidence that ApoE regulates extracellular Abeta metabolism in vivo. Neuron 41:193–202

55. DeMattos RB, O'dell MA, Parsadanian M, Taylor JW, Harmony JAK, Bales KR, et al. (2002) Clusterin promotes amyloid plaque formation and is critical for neuritic toxicity in a mouse model of Alzheimer's disease. Proc Natl Acad Sci 99:10843–10848. doi: 10.1073/pnas.162228299

56. Dermentzaki G, Paschalidis N, Politis PK, Stefanis L (2016) Complex Effects of the ZSCAN21 Transcription Factor on Transcriptional Regulation of α-Synuclein in Primary Neuronal Cultures and *in Vivo*. J Biol Chem 291:8756–8772. doi: 10.1074/jbc.M115.704973

57. Desikan RS, Schork AJ, Wang Y, Thompson WK, Dehghan A, Ridker PM, et al. (2015) Polygenic Overlap Between C-Reactive Protein, Plasma Lipids, and Alzheimer Disease. Circulation 131:2061–2069. doi: 10.1161/CIRCULATIONAHA.115.015489

58. Dourlen P, Fernandez-Gomez FJ, Dupont C, Grenier-Boley B, Bellenguez C, Obriot H, et al. (2017) Functional screening of Alzheimer risk loci identifies PTK2B as an in vivo modulator and early marker of Tau pathology. Mol Psychiatry 22:874–883. doi: 10.1038/mp.2016.59

59. Dourlen P, Fernandez-Gomez FJ, Dupont C, Grenier-Boley B, Bellenguez C, Obriot H, et al. (2017) Functional screening of Alzheimer risk loci identifies PTK2B as an in vivo modulator and early marker of Tau pathology. Mol Psychiatry 22:874–883. doi: 10.1038/mp.2016.59

60. Dräger NM, Nachman E, Winterhoff M, Brühmann S, Shah P, Katsinelos T, et al. (2017) Bin1 directly remodels actin dynamics through its BAR domain. EMBO Rep 18:2051–2066. doi: 10.15252/embr.201744137

61. Dustin ML, Olszowy MW, Holdorf AD, Li J, Bromley S, Desai N, et al. (1998) A novel adaptor protein orchestrates receptor patterning and cytoskeletal polarity in T-cell contacts. Cell 94:667–77

62. Eggert S, Gonzalez AC, Thomas C, Schilling S, Schwarz SM, Tischer C, et al. (2018) Dimerization leads to changes in APP (amyloid precursor protein) trafficking mediated by LRP1 and SorLA. Cell Mol Life Sci 75:301–322. doi: 10.1007/s00018-017-2625-7

63. Fernández-Monreal M, Sánchez-Castillo C, Esteban JA (2016) APPL1 gates long-term potentiation through its plekstrin homology domain. J Cell Sci 129:2793–2803. doi: 10.1242/jcs.183475

64. Fiala M, Lin J, Ringman J, Kermani-Arab V, Tsao G, Patel A, et al. (2005) Ineffective phagocytosis of amyloid-beta by macrophages of Alzheimer's disease patients. J Alzheimers Dis 7:221–32; discussion 255–62

65. Fine D, Flusser H, Markus B, Shorer Z, Gradstein L, Khateeb S, et al. (2015) A syndrome of congenital microcephaly, intellectual disability and dysmorphism with a homozygous mutation in FRMD4A. Eur J Hum Genet 23:1729–1734. doi: 10.1038/ejhg.2014.241

66. Ford JW, McVicar DW (2009) TREM and TREM-like receptors in inflammation and disease. Curr Opin Immunol 21:38–46. doi: 10.1016/j.coi.2009.01.009

67. Forestier A, Douki T, De Rosa V, Béal D, Rachidi W (2015) Combination of Aβ Secretion and Oxidative Stress in an Alzheimer-Like Cell Line Leads to the Over-Expression of the Nucleotide Excision Repair Proteins DDB2 and XPC. Int J Mol Sci 16:17422–17444. doi: 10.3390/ijms160817422

68. Fu AKY, Hung K-W, Huang H, Gu S, Shen Y, Cheng EYL, et al. (2014) Blockade of EphA4 signaling ameliorates hippocampal synaptic dysfunctions in mouse models of Alzheimer's disease. Proc Natl Acad Sci 111:9959–9964. doi: 10.1073/pnas.1405803111

69. Fu Y, Hsiao J-HT, Paxinos G, Halliday GM, Kim WS (2016) ABCA7 Mediates Phagocytic Clearance of Amyloid-β in the Brain. J Alzheimer's Dis 54:569–584. doi: 10.3233/JAD-160456

70. Gallo J-M, Spickett C (2010) The role of CELF proteins in neurological disorders. RNA Biol 7:474–479. doi: 10.4161/rna.7.4.12345

71. García-Cazorla A, Oyarzabal A, Fort J, Robles C, Castejón E, Ruiz-Sala P, et al. (2014) Two Novel Mutations in the *BCKDK* (Branched-Chain Keto-Acid Dehydrogenase Kinase) Gene Are Responsible for a Neurobehavioral Deficit in Two Pediatric Unrelated Patients. Hum Mutat 35:470–477. doi: 10.1002/humu.22513

72. Giralt A, Brito V, Chevy Q, Simonnet C, Otsu Y, Cifuentes-Díaz C, et al. (2017) Pyk2 modulates hippocampal excitatory synapses and contributes to cognitive deficits in a Huntington's disease model. Nat Commun 8:15592. doi: 10.1038/ncomms15592

73. Giralt A, Coura R, Girault J-A (2016) Pyk2 is essential for astrocytes mobility following brain lesion. Glia 64:620–634. doi: 10.1002/glia.22952

74. Giralt A, de Pins B, Cifuentes-Díaz C, López-Molina L, Farah AT, Tible M, et al. (2018) PTK2B/Pyk2 overexpression improves a mouse model of Alzheimer's disease. Exp Neurol 307:62–73. doi: 10.1016/j.expneurol.2018.05.020

75. Girault JA, Costa A, Derkinderen P, Studler JM, Toutant M (1999) FAK and PYK2/CAKbeta in the nervous system: a link between neuronal activity, plasticity and survival? Trends Neurosci 22:257–63

76. Glanz VY, Myasoedova VA, Grechko A V., Orekhov AN (2019) Sialidase activity in human pathologies. Eur J Pharmacol 842:345–350. doi: 10.1016/j.ejphar.2018.11.014

77. Griciuc A, Serrano-Pozo A, Parrado AR, Lesinski AN, Asselin CN, Mullin K, et al. (2013) Alzheimer's Disease Risk Gene CD33 Inhibits Microglial Uptake of Amyloid Beta. Neuron 78:631–643. doi: 10.1016/j.neuron.2013.04.014

78. Le Guennec K, Nicolas G, Quenez O, Charbonnier C, Wallon D, Bellenguez C, et al. (2016) *ABCA7* rare variants and Alzheimer disease risk. Neurology 86:2134–2137. doi: 10.1212/WNL.0000000000002627

79. Guerreiro R, Wojtas A, Bras J, Carrasquillo M, Rogaeva E, Majounie E, et al. (2013) *TREM2* Variants in Alzheimer's Disease. N Engl J Med 368:117–127. doi: 10.1056/NEJMoa1211851

80. Gurses MS, Ural MN, Gulec MA, Akyol O, Akyol S (2016) Pathophysiological Function of ADAMTS Enzymes on Molecular Mechanism of Alzheimer's Disease. Aging Dis 7:479. doi: 10.14336/AD.2016.0111

81. Haas LT, Salazar S V., Kostylev MA, Um JW, Kaufman AC, Strittmatter SM (2016) Metabotropic glutamate receptor 5 couples cellular prion protein to intracellular signalling in Alzheimer's disease. Brain 139:526–546. doi: 10.1093/brain/awv356

82. Haass C, Kaether C, Thinakaran G, Sisodia S (2012) Trafficking and proteolytic processing of APP. Cold Spring Harb Perspect Med 2:a006270. doi: 10.1101/cshperspect.a006270

83. Haglund K, Nezis IP, Lemus D, Grabbe C, Wesche J, Liestøl K, et al. (2010) Cindr Interacts with Anillin to Control Cytokinesis in Drosophila melanogaster. Curr Biol 20:944–950. doi: 10.1016/j.cub.2010.03.068

84. Hammad SM, Ranganathan S, Loukinova E, Twal WO, Argraves WS (1997) Interaction of apolipoprotein J-amyloid beta-peptide complex with low density lipoprotein receptor-related protein-2/megalin. A mechanism to prevent pathological accumulation of amyloid beta-peptide. J Biol Chem 272:18644–9

85. Han S, Nam J, Li Y, Kim S, Cho S-H, Cho YS, et al. (2010) Regulation of Dendritic Spines, Spatial Memory, and Embryonic Development by the TANC Family of PSD-95-Interacting Proteins. J Neurosci 30:15102–15112. doi: 10.1523/JNEUROSCI.3128-10.2010

86. Haney MS, Bohlen CJ, Morgens DW, Ousey JA, Barkal AA, Tsui CK, et al. (2018) Identification of phagocytosis regulators using magnetic genome-wide CRISPR screens. Nat Genet 50:1716–1727. doi: 10.1038/s41588-018-0254-1

87. Hardaway JA, Sturgeon SM, Snarrenberg CL, Li Z, Xu XZS, Bermingham DP, et al. (2015) Glial Expression of the Caenorhabditis elegans Gene swip-10 Supports Glutamate Dependent Control of Extrasynaptic Dopamine Signaling. J Neurosci 35:9409–9423. doi: 10.1523/JNEUROSCI.0800-15.2015

88. Harel A, Mattson MP, Yao PJ (2011) CALM, A Clathrin Assembly Protein, Influences Cell Surface GluR2 Abundance. NeuroMolecular Med 13:88–90. doi: 10.1007/s12017-010-8142-6

89. Harel A, Wu F, Mattson MP, Morris CM, Yao PJ (2008) Evidence for CALM in Directing VAMP2 Trafficking. Traffic 9:417–429. doi: 10.1111/j.1600-0854.2007.00694.x

90. Harold D, Abraham R, Hollingworth P, Sims R, Gerrish A, Hamshere ML, et al. (2009) Genome-wide association study identifies variants at CLU and PICALM associated with Alzheimer's disease. Nat Genet 41:1088–93. doi: 10.1038/ng.440

91. Harrison BJ, Venkat G, Lamb JL, Hutson TH, Drury C, Rau KK, et al. (2016) The Adaptor Protein CD2AP Is a Coordinator of Neurotrophin Signaling-Mediated Axon Arbor Plasticity. J Neurosci 36:4259–75. doi: 10.1523/JNEUROSCI.2423-15.2016

92. He F, Umehara T, Saito K, Harada T, Watanabe S, Yabuki T, et al. (2010) Structural Insight into the Zinc Finger CW Domain as a Histone Modification Reader. Structure 18:1127–1139. doi: 10.1016/j.str.2010.06.012

93. Hemming ML, Selkoe DJ (2005) Amyloid beta-protein is degraded by cellular angiotensin-converting enzyme (ACE) and elevated by an ACE inhibitor. J Biol Chem 280:37644–50. doi: 10.1074/jbc.M508460200

94. Hirai H, Maru Y, Hagiwara K, Nishida J, Takaku F (1987) A novel putative tyrosine kinase receptor encoded by the eph gene. Science 238:1717–20

95. Hollingworth P, Harold D, Sims R, Gerrish A, Lambert J-C, Carrasquillo MM, et al. (2011) Common variants at ABCA7, MS4A6A/MS4A4E, EPHA1, CD33 and CD2AP are associated with Alzheimer's disease. Nat Genet 43:429–35. doi: 10.1038/ng.803

96. Howell MD, Torres-Collado AX, Iruela-Arispe ML, Gottschall PE (2012) Selective Decline of Synaptic Protein Levels in the Frontal Cortex of Female Mice Deficient in the Extracellular Metalloproteinase ADAMTS1. PLoS One 7:e47226. doi: 10.1371/journal.pone.0047226

97. Hsin H, Kim MJ, Wang C-F, Sheng M (2010) Proline-Rich Tyrosine Kinase 2 Regulates Hippocampal Long-Term Depression. J Neurosci 30:11983–11993. doi: 10.1523/JNEUROSCI.1029-10.2010

98. Hu J, Huang T, Li T, Guo Z, Cheng L (2012) c-Maf Is Required for the Development of Dorsal Horn Laminae III/IV Neurons and Mechanoreceptive DRG Axon Projections. J Neurosci 32:5362–5373. doi: 10.1523/JNEUROSCI.6239-11.2012

99. Hu J, Igarashi A, Kamata M, Nakagawa H (2001) Angiotensin-converting enzyme degrades Alzheimer amyloid beta-peptide (A beta); retards A beta aggregation, deposition, fibril formation; and inhibits cytotoxicity. J Biol Chem 276:47863–8. doi: 10.1074/jbc.M104068200

100. Huang K-L, Marcora E, Pimenova AA, Di Narzo AF, Kapoor M, Jin SC, et al. (2017) A common haplotype lowers PU.1 expression in myeloid cells and delays onset of Alzheimer's disease. Nat Neurosci 20:1052–1061. doi: 10.1038/nn.4587

101. Huang TY, Zhao Y, Jiang L, Li X, Liu Y, Sun Y, et al. (2017) SORLA attenuates EphA4 signaling and amyloid β–induced neurodegeneration. J Exp Med 214:3669–3685. doi: 10.1084/jem.20171413

102. Huang Y, Lu W, Ali DW, Pelkey KA, Pitcher GM, Lu YM, et al. (2001) CAKbeta/Pyk2 kinase is a signaling link for induction of long-term potentiation in CA1 hippocampus. Neuron 29:485–96

103. Huynh T-P V., Davis AA, Ulrich JD, Holtzman DM (2017) Apolipoprotein E and Alzheimer's disease: the influence of apolipoprotein E on amyloid-β and other amyloidogenic proteins. J Lipid Res 58:824–836. doi: 10.1194/jlr.R075481

104. Ikezu T, Chen C, DeLeo AM, Zeldich E, Fallin MD, Kanaan NM, et al. (2018) Tau Phosphorylation is Impacted by Rare AKAP9 Mutations Associated with Alzheimer Disease in African Americans. J Neuroimmune Pharmacol 13:254–264. doi: 10.1007/s11481-018-9781-x

105. Inoue E, Deguchi-Tawarada M, Togawa A, Matsui C, Arita K, Katahira-Tayama S, et al. (2009) Synaptic activity prompts γ-secretase–mediated cleavage of EphA4 and dendritic spine formation. J Cell Biol 185:551–564. doi: 10.1083/jcb.200809151

106. Ivankovic-Dikic I, Grönroos E, Blaukat A, Barth BU, Dikic I (2000) Pyk2 and FAK regulate neurite outgrowth induced by growth factors and integrins. Nat Cell Biol 2:574–81. doi: 10.1038/35023515

107. Jakobsdottir J, van der Lee SJ, Bis JC, Chouraki V, Li-Kroeger D, Yamamoto S, et al. (2016) Rare Functional Variant in TM2D3 is Associated with Late-Onset Alzheimer's Disease. PLOS Genet 12:e1006327. doi: 10.1371/journal.pgen.1006327

108. Jansen IE, Savage JE, Watanabe K, Bryois J, Williams DM, Steinberg S, et al. (2019) Genome-wide meta-analysis identifies new loci and functional pathways influencing Alzheimer's disease risk. Nat Genet. doi: 10.1038/s41588-018-0311-9

109. Jay TR, Miller CM, Cheng PJ, Graham LC, Bemiller S, Broihier ML, et al. (2015) TREM2 deficiency eliminates TREM2 $^{+}$ inflammatory macrophages and ameliorates pathology in Alzheimer's disease mouse models. J Exp Med 212:287–295. doi: 10.1084/jem.20142322

110. Jehle AW, Gardai SJ, Li S, Linsel-Nitschke P, Morimoto K, Janssen WJ, et al. (2006) ATP-binding cassette transporter A7 enhances phagocytosis of apoptotic cells and associated ERK signaling in macrophages. J Cell Biol 174:547–556. doi: 10.1083/jcb.200601030

111. Jiang T, Tan L, Zhu X-C, Zhang Q-Q, Cao L, Tan M-S, et al. (2014) Upregulation of TREM2 Ameliorates Neuropathology and Rescues Spatial Cognitive Impairment in a Transgenic Mouse Model of Alzheimer's Disease. Neuropsychopharmacology 39:2949–2962. doi: 10.1038/npp.2014.164

112. Jiang T, Tan L, Zhu X-C, Zhou J-S, Cao L, Tan M-S, et al. (2015) Silencing of TREM2 exacerbates tau pathology, neurodegenerative changes, and spatial learning deficits in P301S tau transgenic mice. Neurobiol Aging 36:3176–3186. doi: 10.1016/j.neurobiolaging.2015.08.019

113. Jiang T, Zhang Y-D, Gao Q, Ou Z, Gong P-Y, Shi J-Q, et al. (2018) TREM2 Ameliorates Neuronal Tau Pathology Through Suppression of Microglial Inflammatory Response. Inflammation 41:811–823. doi: 10.1007/s10753-018-0735-5

114. Jin SC, Benitez BA, Karch CM, Cooper B, Skorupa T, Carrell D, et al. (2014) Coding variants in TREM2 increase risk for Alzheimer's disease. Hum Mol Genet 23:5838–5846. doi: 10.1093/hmg/ddu277

115. Jochemsen HM, Teunissen CE, Ashby EL, van der Flier WM, Jones RE, Geerlings MI, et al. (2014) The association of angiotensin-converting enzyme with biomarkers for Alzheimer's disease. Alzheimers Res Ther 6:27. doi: 10.1186/alzrt257

116. Johnson RI, Seppa MJ, Cagan RL (2008) The *Drosophila* CD2AP/CIN85 orthologue Cindr regulates junctions and cytoskeleton dynamics during tissue patterning. J Cell Biol 180:1191–1204. doi: 10.1083/jcb.200706108

117. Jonsson T, Stefansson H, Steinberg S, Jonsdottir I, Jonsson P V, Snaedal J, et al. (2013) Variant of TREM2 associated with the risk of Alzheimer's disease. N Engl J Med 368:107–16. doi: 10.1056/NEJMoa1211103

118. Jun G, Ibrahim-Verbaas CA, Vronskaya M, Lambert J-C, Chung J, Naj AC, et al. (2016) A novel Alzheimer disease locus located near the gene encoding tau protein. Mol Psychiatry 21:108–117. doi: 10.1038/mp.2015.23

119. Jun GR, Chung J, Mez J, Barber R, Beecham GW, Bennett DA, et al. (2017) Transethnic genome-wide scan identifies novel Alzheimer's disease loci. Alzheimer's Dement 13:727–738. doi: 10.1016/j.jalz.2016.12.012

120. Kanatsu K, Hori Y, Takatori S, Watanabe T, Iwatsubo T, Tomita T (2016) Partial loss of CALM function reduces Aβ42 production and amyloid deposition *in vivo*. Hum Mol Genet 25:3988–3997. doi: 10.1093/hmg/ddw239

121. Kanatsu K, Morohashi Y, Suzuki M, Kuroda H, Watanabe T, Tomita T, et al. (2014) Decreased CALM expression reduces Aβ42 to total Aβ ratio through clathrin-mediated endocytosis of γ-secretase. Nat Commun 5:3386. doi: 10.1038/ncomms4386

122. Karch CM, Ezerskiy LA, Bertelsen S, Goate AM, Goate AM (2016) Alzheimer's Disease Risk Polymorphisms Regulate Gene Expression in the ZCWPW1 and the CELF1 Loci. PLoS One 11:e0148717. doi: 10.1371/journal.pone.0148717

123. Karch CM, Goate AM (2014) Alzheimer's Disease Risk Genes and Mechanisms of Disease Pathogenesis. Biol Psychiatry 77:43–51. doi: 10.1016/j.biopsych.2014.05.006

124. Karch CM, Jeng AT, Nowotny P, Cady J, Cruchaga C, Goate AM (2012) Expression of novel Alzheimer's disease risk genes in control and Alzheimer's disease brains. PLoS One 7:e50976. doi: 10.1371/journal.pone.0050976

125. Kaufman AC, Salazar S V, Haas LT, Yang J, Kostylev MA, Jeng AT, et al. (2015) Fyn inhibition rescues established memory and synapse loss in Alzheimer mice. Ann Neurol 77:953–971. doi: 10.1002/ana.24394

126. Kauwe JSK, Bailey MH, Ridge PG, Perry R, Wadsworth ME, Hoyt KL, et al. (2014) Genome-Wide Association Study of CSF Levels of 59 Alzheimer's Disease Candidate Proteins: Significant Associations with Proteins Involved in Amyloid Processing and Inflammation. PLoS Genet 10:e1004758. doi: 10.1371/journal.pgen.1004758

127. Kehoe PG (2018) The Coming of Age of the Angiotensin Hypothesis in Alzheimer's Disease: Progress Toward Disease Prevention and Treatment? J Alzheimers Dis 62:1443–1466. doi: 10.3233/JAD-171119

128. Killick R, Ribe EM, Al-Shawi R, Malik B, Hooper C, Fernandes C, et al. (2014) Clusterin regulates β-amyloid toxicity via Dickkopf-1-driven induction of the wnt-PCP-JNK pathway. Mol Psychiatry 19:88–98. doi: 10.1038/mp.2012.163

129. Kim M, Semple I, Kim B, Kiers A, Nam S, Park H-W, et al. (2015) Drosophila Gyf/GRB10 interacting GYF protein is an autophagy regulator that controls neuron and muscle homeostasis. Autophagy 11:1358–72. doi: 10.1080/15548627.2015.1063766

130. Kim M, Suh J, Romano D, Truong MH, Mullin K, Hooli B, et al. (2009) Potential late-onset Alzheimer's disease-associated mutations in the ADAM10 gene attenuate {alpha}-secretase activity. Hum Mol Genet 18:3987–96. doi: 10.1093/hmg/ddp323

131. Kim M, Wende H, Walcher J, Kühnemund J, Cheret C, Kempa S, et al. (2018) Maf links Neuregulin1 signaling to cholesterol synthesis in myelinating Schwann cells. Genes Dev 32:645–657. doi: 10.1101/gad.310490.117

132. Kim S, Sato Y, Mohan PS, Peterhoff C, Pensalfini A, Rigoglioso A, et al. (2016) Evidence that the rab5 effector APPL1 mediates APP-βCTF-induced dysfunction of endosomes in Down syndrome and Alzheimer's disease. Mol Psychiatry 21:707–716. doi: 10.1038/mp.2015.97

133. Kim WS, Li H, Ruberu K, Chan S, Elliott DA, Low JK, et al. (2013) Deletion of Abca7 Increases Cerebral Amyloid- Accumulation in the J20 Mouse Model of Alzheimer's Disease. J Neurosci 33:4387–4394. doi: 10.1523/JNEUROSCI.4165-12.2013

134. Kim WS, Weickert CS, Garner B (2008) Role of ATP-binding cassette transporters in brain lipid transport and neurological disease. J Neurochem 104:1145–1166. doi: 10.1111/j.1471-4159.2007.05099.x

135. Kirsch KH, Georgescu MM, Ishimaru S, Hanafusa H (1999) CMS: an adapter molecule involved in cytoskeletal rearrangements. Proc Natl Acad Sci U S A 96:6211–6

136. Kleinberger G, Yamanishi Y, Suarez-Calvet M, Czirr E, Lohmann E, Cuyvers E, et al. (2014) TREM2 mutations implicated in neurodegeneration impair cell surface transport and phagocytosis. Sci Transl Med 6:243ra86–243ra86. doi: 10.1126/scitranslmed.3009093

137. Kobayashi S, Sawano A, Nojima Y, Shibuya M, Maru Y (2004) The c-Cbl/CD2AP complex regulates VEGF-induced endocytosis and degradation of Flt-1 (VEGFR-1). FASEB J 18:929–31. doi: 10.1096/fj.03-0767fje

138. Kunkle BW, Grenier-Boley B, Sims R, Bis JC, Naj AC, Boland A, et al. (2018) Meta-analysis of genetic association with diagnosed Alzheimer's disease identifies novel risk loci and implicates Abeta, Tau, immunity and lipid processing. bioRxiv

139. Kurosawa N, Chen GY, Kadomatsu K, Ikematsu S, Sakuma S, Muramatsu T (2001) Glypican-2 binds to midkine: the role of glypican-2 in neuronal cell adhesion and neurite outgrowth. Glycoconj J 18:499–507

140. Lai K-O, Ip NY (2009) Synapse development and plasticity: roles of ephrin/Eph receptor signaling. Curr Opin Neurobiol 19:275–283. doi: 10.1016/j.conb.2009.04.009

141. Lai-Cheong JE, Parsons M, McGrath JA (2010) The role of kindlins in cell biology and relevance to human disease. Int J Biochem Cell Biol 42:595–603. doi: 10.1016/j.biocel.2009.10.015

142. Lambert J-C, Grenier-Boley B, Harold D, Zelenika D, Chouraki V, Kamatani Y, et al. (2013) Genome-wide haplotype association study identifies the FRMD4A gene as a risk locus for Alzheimer's disease. Mol Psychiatry 18:461–70. doi: 10.1038/mp.2012.14

143. Lambert J-C, Heath S, Even G, Campion D, Sleegers K, Hiltunen M, et al. (2009) Genome-wide association study identifies variants at CLU and CR1 associated with Alzheimer's disease. Nat Genet 41:1094–9. doi: 10.1038/ng.439

144. Lambert JC, Ibrahim-Verbaas CA, Harold D, Naj AC, Sims R, Bellenguez C, et al. (2013) Meta-analysis of 74,046 individuals identifies 11 new susceptibility loci for Alzheimer's disease. Nat Genet 45:1452–8. doi: 10.1038/ng.2802

145. Lanzetti L, Palamidessi A, Areces L, Scita G, Di Fiore PP (2004) Rab5 is a signalling GTPase involved in actin remodelling by receptor tyrosine kinases. Nature 429:309–314. doi: 10.1038/nature02542

146. Lanzetti L, Rybin V, Malabarba MG, Christoforidis S, Scita G, Zerial M, et al. (2000) The Eps8 protein coordinates EGF receptor signalling through Rac and trafficking through Rab5. Nature 408:374–377. doi: 10.1038/35042605

147. Lassot I, Mora S, Lesage S, Zieba BA, Coque E, Condroyer C, et al. (2018) The E3 Ubiquitin Ligases TRIM17 and TRIM41 Modulate α-Synuclein Expression by Regulating ZSCAN21. Cell Rep 25:2484–2496.e9. doi: 10.1016/j.celrep.2018.11.002

148. Lee S, Salazar S V., Cox TO, Strittmatter SM (2019) Pyk2 Signaling through Graf1 and RhoA GTPase is Required for Amyloid-β Oligomer Triggered Synapse Loss. J Neurosci 2983–18. doi: 10.1523/JNEUROSCI.2983-18.2018

149. Lessard CB, Malnik SL, Zhou Y, Ladd TB, Cruz PE, Ran Y, et al. (2018) High-affinity interactions and signal transduction between Aβ oligomers and TREM2. EMBO Mol Med 10:e9027. doi: 10.15252/emmm.201809027

150. Li X-F, Kraev AS, Lytton J (2002) Molecular Cloning of a Fourth Member of the Potassium-dependent Sodium-Calcium Exchanger Gene Family, NCKX4. J Biol Chem 277:48410–48417. doi: 10.1074/jbc.M210011200

151. Li X-F, Lytton J (2014) An Essential Role for the K^+ -dependent Na^+ /Ca^{2+} -exchanger, NCKX4, in Melanocortin-4-receptor-dependent Satiety. J Biol Chem 289:25445–25459. doi: 10.1074/jbc.M114.564450

152. Liao F, Jiang H, Srivatsan S, Xiao Q, Lefton KB, Yamada K, et al. (2015) Effects of CD2-associated protein deficiency on amyloid-β in neuroblastoma cells and in an APP transgenic mouse model. Mol Neurodegener 10:12. doi: 10.1186/s13024-015-0006-y

153. Liu JZ, Erlich Y, Pickrell JK (2017) Case-control association mapping by proxy using family history of disease. Nat Genet 49:325–331. doi: 10.1038/ng.3766

154. Logue MW, Schu M, Vardarajan BN, Farrell J, Bennett DA, Buxbaum JD, et al. (2014) Two rare AKAP9 variants are associated with Alzheimer's disease in African Americans. Alzheimer's Dement 10:609–618.e11. doi: 10.1016/j.jalz.2014.06.010

155. Lu C, Chen J, Xu H, Zhou X, He Q, Li Y, et al. (2014) MIR106B and MIR93 Prevent Removal of Bacteria From Epithelial Cells by Disrupting ATG16L1-Mediated Autophagy. Gastroenterology 146:188–199. doi: 10.1053/j.gastro.2013.09.006

156. Luo S, Schaefer AM, Dour S, Nonet ML (2014) The conserved LIM domain-containing focal adhesion protein ZYX-1 regulates synapse maintenance in Caenorhabditis elegans. Development 141:3922–3933. doi: 10.1242/dev.108217

157. Lynch DK, Winata SC, Lyons RJ, Hughes WE, Lehrbach GM, Wasinger V, et al. (2003) A Cortactin-CD2-associated protein (CD2AP) complex provides a novel link between epidermal growth factor receptor endocytosis and the actin cytoskeleton. J Biol Chem 278:21805–13. doi: 10.1074/jbc.M211407200

158. Ma J, Yu J-T, Tan L (2015) MS4A Cluster in Alzheimer's Disease. Mol Neurobiol 51:1240–1248. doi: 10.1007/s12035-014-8800-z

159. Mahmoudi R, Kisserli A, Novella J-L, Donvito B, Dramé M, Réveil B, et al. (2015) Alzheimer's disease is associated with low density of the long CR1 isoform. Neurobiol Aging 36:1766.e5–1766.e12. doi: 10.1016/j.neurobiolaging.2015.01.006

160. Majumdar D, Nebhan CA, Hu L, Anderson B, Webb DJ (2011) An APPL1/Akt signaling complex regulates dendritic spine and synapse formation in hippocampal neurons. Mol Cell Neurosci 46:633–644. doi: 10.1016/j.mcn.2011.01.003

161. Malik M, Parikh I, Vasquez JB, Smith C, Tai L, Bu G, et al. (2015) Genetics ignite focus on microglial inflammation in Alzheimer's disease. Mol Neurodegener 10:52. doi: 10.1186/s13024-015-0048-1

162. Malik M, Simpson JF, Parikh I, Wilfred BR, Fardo DW, Nelson PT, et al. (2013) CD33 Alzheimer's Risk-Altering Polymorphism, CD33 Expression, and Exon 2 Splicing. J Neurosci 33:13320–13325. doi: 10.1523/JNEUROSCI.1224-13.2013

163. Malki I, Cantrelle FX, Sottejeau Y, Lippens G, Lambert JC, Landrieu I (2017) Regulation of the interaction between the neuronal BIN1 isoform 1 and Tau proteins – role of the SH3 domain. FEBS J 284:3218–3229. doi: 10.1111/febs.14185

164. Mallaret M, Synofzik M, Lee J, Sagum CA, Mahajnah M, Sharkia R, et al. (2014) The tumour suppressor gene WWOX is mutated in autosomal recessive cerebellar ataxia with epilepsy and mental retardation. Brain 137:411–419. doi: 10.1093/brain/awt338

165. Mannan AU, Roussa E, Kraus C, Rickmann M, Maenner J, Nayernia K, et al. (2004) Mutation in the gene encoding lysosomal acid phosphatase (Acp2) causes cerebellum and skin malformation in mouse. Neurogenetics 5:229–238. doi: 10.1007/s10048-004-0197-9

166. Marques CA, Keil U, Bonert A, Steiner B, Haass C, Müller WE, et al. (2003) Neurotoxic Mechanisms Caused by the Alzheimer's Disease-linked Swedish Amyloid Precursor Protein Mutation. J Biol Chem 278:28294–28302. doi: 10.1074/jbc.M212265200

167. Martinu L, Santiago-Walker A, Qi H, Chou MM (2002) Endocytosis of Epidermal Growth Factor Receptor Regulated by Grb2-mediated Recruitment of the Rab5 GTPase-activating Protein RN-tre. J Biol Chem 277:50996–51002. doi: 10.1074/jbc.M204869200

168. Martiskainen H, Viswanathan J, Nykänen NP, Kurki M, Helisalmi S, Natunen T, et al. (2015) Transcriptomics and mechanistic elucidation of Alzheimer's disease risk genes in the brain and invitro models. Neurobiol Aging 36:1221.e15–1221.e28. doi: 10.1016/j.neurobiolaging.2014.09.003

169. Matsui C, Inoue E, Kakita A, Arita K, Deguchi-Tawarada M, Togawa A, et al. (2012) Involvement of the γ-Secretase-Mediated EphA4 Signaling Pathway in Synaptic Pathogenesis of Alzheimer's Disease. Brain Pathol 22:776–787. doi: 10.1111/j.1750-3639.2012.00587.x

170. Matsuo A, Walker DG, Terai K, McGeer PL (1996) Expression of CD43 in human microglia and its downregulation in Alzheimer's disease. J Neuroimmunol 71:81–6

171. May PC, Lampert-Etchells M, Johnson SA, Poirier J, Masters JN, Finch CE (1990) Dynamics of gene expression for a hippocampal glycoprotein elevated in Alzheimer's disease and in response to experimental lesions in rat. Neuron 5:831–9

172. McGeer PL, Itagaki S, Tago H, McGeer EG (1987) Reactive microglia in patients with senile dementia of the Alzheimer type are positive for the histocompatibility glycoprotein HLA-DR. Neurosci Lett 79:195–200

173. McNally KE, Faulkner R, Steinberg F, Gallon M, Ghai R, Pim D, et al. (2017) Retriever is a multiprotein complex for retromer-independent endosomal cargo recycling. Nat Cell Biol 19:1214–1225. doi: 10.1038/ncb3610

174. Mentrup T, Fluhrer R, Schröder B (2017) Latest emerging functions of SPP/SPPL intramembrane proteases. Eur J Cell Biol 96:372–382. doi: 10.1016/j.ejcb.2017.03.002

175. Messina S, Onofri F, Bongiorno-Borbone L, Giovedì S, Valtorta F, Girault JA, et al. (2003) Specific interactions of neuronal focal adhesion kinase isoforms with Src kinases and amphiphysin. J Neurochem 84:253–265. doi: 10.1046/j.1471-4159.2003.01519.x

176. Miguel RF, Pollak A, Lubec G (2005) Metalloproteinase ADAMTS-1 but not ADAMTS-5 is manifold overexpressed in neurodegenerative disorders as Down syndrome, Alzheimer's and Pick's disease. Brain Res Mol Brain Res 133:1–5. doi: 10.1016/j.molbrainres.2004.09.008

177. Miller SE, Sahlender DA, Graham SC, Höning S, Robinson MS, Peden AA, et al. (2011) The Molecular Basis for the Endocytosis of Small R-SNAREs by the Clathrin Adaptor CALM. Cell 147:1118–1131. doi: 10.1016/j.cell.2011.10.038

178. Miners JS, Clarke P, Love S (2017) Clusterin levels are increased in Alzheimer's disease and influence the regional distribution of Aβ. Brain Pathol 27:305–313. doi: 10.1111/bpa.12392

179. Miners S, Ashby E, Baig S, Harrison R, Tayler H, Speedy E, et al. (2009) Angiotensin-converting enzyme levels and activity in Alzheimer's disease: differences in brain and CSF ACE and association with ACE1 genotypes. Am J Transl Res 1:163–77

180. Miyagawa T, Ebinuma I, Morohashi Y, Hori Y, Young Chang M, Hattori H, et al. (2016) BIN1 regulates BACE1 intracellular trafficking and amyloid-β production. Hum Mol Genet 25:2948–2958. doi: 10.1093/hmg/ddw146

181. Mo Y, Williams C, Miller CA (2012) DENN/MADD/IG20 Alternative Splicing Changes and Cell Death in Alzheimer's Disease. J Mol Neurosci 48:97–110. doi: 10.1007/s12031-012-9782-9

182. Moreau K, Fleming A, Imarisio S, Lopez Ramirez A, Mercer JL, Jimenez-Sanchez M, et al. (2014) PICALM modulates autophagy activity and tau accumulation. Nat Commun 5:4998. doi: 10.1038/ncomms5998

183. Mukherjee S, Russell JC, Carr DT, Burgess JD, Allen M, Serie DJ, et al. (2017) Systems biology approach to late-onset Alzheimer's disease genome-wide association study identifies novel candidate genes validated using brain expression data and Caenorhabditis elegans experiments. Alzheimer's Dement 13:1133–1142. doi: 10.1016/j.jalz.2017.01.016

184. Naj AC, Jun G, Beecham GW, Wang L-S, Vardarajan BN, Buros J, et al. (2011) Common variants at MS4A4/MS4A6E, CD2AP, CD33 and EPHA1 are associated with late-onset Alzheimer's disease. Nat Genet 43:436–41. doi: 10.1038/ng.801

185. Narayan P, Orte A, Clarke RW, Bolognesi B, Hook S, Ganzinger KA, et al. (2011) The extracellular chaperone clusterin sequesters oligomeric forms of the amyloid-β(1-40) peptide. Nat Struct Mol Biol 19:79–83. doi: 10.1038/nsmb.2191

186. Neve RL, Coopersmith R, McPhie DL, Santeufemio C, Pratt KG, Murphy CJ, et al. (1998) The neuronal growth-associated protein GAP-43 interacts with rabaptin-5 and participates in endocytosis. J Neurosci 18:7757–67

187. Nicolas G, Charbonnier C, Wallon D, Quenez O, Bellenguez C, Grenier-Boley B, et al. (2016) SORL1 rare variants: a major risk factor for familial early-onset Alzheimer's disease. Mol Psychiatry 21:831–836. doi: 10.1038/mp.2015.121

188. Nolz JC, Gomez TS, Zhu P, Li S, Medeiros RB, Shimizu Y, et al. (2006) The WAVE2 Complex Regulates Actin Cytoskeletal Reorganization and CRAC-Mediated Calcium Entry during T Cell Activation. Curr Biol 16:24–34. doi: 10.1016/j.cub.2005.11.036

189. Novarino G, El-Fishawy P, Kayserili H, Meguid NA, Scott EM, Schroth J, et al. (2012) Mutations in BCKD-kinase Lead to a Potentially Treatable Form of Autism with Epilepsy. Science (80-) 338:394–397. doi: 10.1126/science.1224631

190. Nuutinen T, Suuronen T, Kauppinen A, Salminen A (2009) Clusterin: A forgotten player in Alzheimer's disease. Brain Res Rev 61:89–104. doi: 10.1016/j.brainresrev.2009.05.007

191. O'Nuallain B, Acero L, Williams AD, Koeppen HPM, Weber A, Schwarz HP, et al. (2008) Human plasma contains cross-reactive Abeta conformer-specific IgG antibodies. Biochemistry 47:12254–6. doi: 10.1021/bi801767k

192. Oba R, Igarashi A, Kamata M, Nagata K, Takano S, Nakagawa H (2005) The N-terminal active centre of human angiotensin-converting enzyme degrades Alzheimer amyloid beta-peptide. Eur J Neurosci 21:733–40. doi: 10.1111/j.1460-9568.2005.03912.x

193. Oda T, Wals P, Osterburg HH, Johnson SA, Pasinetti GM, Morgan TE, et al. (1995) Clusterin (apoJ) alters the aggregation of amyloid beta-peptide (A beta 1-42) and forms slowly sedimenting A beta complexes that cause oxidative stress. Exp Neurol 136:22–31

194. Offe K, Dodson SE, Shoemaker JT, Fritz JJ, Gearing M, Levey AI, et al. (2006) The Lipoprotein Receptor LR11 Regulates Amyloid beta Production and Amyloid Precursor Protein Traffic in Endosomal Compartments. J Neurosci 26:1596–1603. doi: 10.1523/JNEUROSCI.4946-05.2006

195. Ogawa A, Yamazaki Y, Nakamori M, Takahashi T, Kurashige T, Hiji M, et al. (2013) Characterization and distribution of adaptor protein containing a PH domain, PTB domain and leucine zipper motif (APPL1) in Alzheimer's disease hippocampus: an immunohistochemical study. Brain Res 1494:118–124. doi: 10.1016/j.brainres.2012.12.010

196. Olave I, Wang W, Xue Y, Kuo A, Crabtree GR (2002) Identification of a polymorphic, neuron-specific chromatin remodeling complex. Genes Dev 16:2509–2517. doi: 10.1101/gad.992102

197. Oyagi A, Hara H (2012) Essential Roles of Heparin-Binding Epidermal Growth Factor-Like Growth Factor in the Brain. CNS Neurosci Ther 18:803–810. doi: 10.1111/j.1755-5949.2012.00371.x

198. Piccio L, Deming Y, Del-Águila JL, Ghezzi L, Holtzman DM, Fagan AM, et al. (2016) Cerebrospinal fluid soluble TREM2 is higher in Alzheimer disease and associated with mutation status. Acta Neuropathol 131:925–933. doi: 10.1007/s00401-016-1533-5

199. Postina R, Schroeder A, Dewachter I, Bohl J, Schmitt U, Kojro E, et al. (2004) A disintegrin-metalloproteinase prevents amyloid plaque formation and hippocampal defects in an Alzheimer disease mouse model. J Clin Invest 113:1456–1464. doi: 10.1172/JCI20864

200. Pozueta J, Lefort R, Ribe EM, Troy CM, Arancio O, Shelanski M (2013) Caspase-2 is required for dendritic spine and behavioural alterations in J20 APP transgenic mice. Nat Commun 4:1939. doi: 10.1038/ncomms2927

201. Prokic I, Cowling BS, Laporte J (2014) Amphiphysin 2 (BIN1) in physiology and diseases. J Mol Med 92:453–463. doi: 10.1007/s00109-014-1138-1

202. Raj T, Ryan KJ, Replogle JM, Chibnik LB, Rosenkrantz L, Tang A, et al. (2014) CD33: increased inclusion of exon 2 implicates the Ig V-set domain in Alzheimer's disease susceptibility. Hum Mol Genet 23:2729–2736. doi: 10.1093/hmg/ddt666

203. Ramirez LM, Goukasian N, Porat S, Hwang KS, Eastman JA, Hurtz S, et al. (2016) Common variants in ABCA7 and MS4A6A are associated with cortical and hippocampal atrophy. Neurobiol Aging 39:82–89. doi: 10.1016/j.neurobiolaging.2015.10.037

204. Ramos de Matos M, Ferreira C, Herukka S-K, Soininen H, Janeiro A, Santana I, et al. (2018) Quantitative Genetics Validates Previous Genetic Variants and Identifies Novel Genetic Players Influencing Alzheimer's Disease Cerebrospinal Fluid Biomarkers. J Alzheimer's Dis 66:639–652. doi: 10.3233/JAD-180512

205. Rangaraju S, Dammer EB, Raza SA, Gao T, Xiao H, Betarbet R, et al. (2018) Quantitative proteomics of acutely-isolated mouse microglia identifies novel immune Alzheimer's disease-related proteins. Mol Neurodegener 13:34. doi: 10.1186/s13024-018-0266-4

206. Rathore N, Ramani SR, Pantua H, Payandeh J, Bhangale T, Wuster A, et al. (2018) Paired Immunoglobulin-like Type 2 Receptor Alpha G78R variant alters ligand binding and confers protection to Alzheimer's disease. PLoS Genet 14:e1007427. doi: 10.1371/journal.pgen.1007427

207. Rogaeva E, Meng Y, Lee JH, Gu Y, Kawarai T, Zou F, et al. (2007) The neuronal sortilin-related receptor SORL1 is genetically associated with Alzheimer disease. Nat Genet 39:168–77. doi: 10.1038/ng1943

208. Rogers J, Li R, Mastroeni D, Grover A, Leonard B, Ahern G, et al. (2006) Peripheral clearance of amyloid β peptide by complement C3-dependent adherence to erythrocytes. Neurobiol Aging 27:1733–1739. doi: 10.1016/j.neurobiolaging.2005.09.043

209. Rosenberger AF, Rozemuller AJ, van der Flier WM, Scheltens P, van der Vies SM, Hoozemans JJ (2014) Altered distribution of the EphA4 kinase in hippocampal brain tissue of patients with Alzheimer's disease correlates with pathology. Acta Neuropathol Commun 2:79. doi: 10.1186/s40478-014-0079-9

210. De Rossi P, Andrew RJ, Musial TF, Buggia-Prevot V, Xu G, Ponnusamy M, et al. (2018) Aberrant accrual of BIN1 near Alzheimer's disease amyloid deposits in transgenic models. Brain Pathol. doi: 10.1111/bpa.12687

211. Rouka E, Simister PC, Janning M, Kumbrink J, Konstantinou T, Muniz JRC, et al. (2015) Differential Recognition Preferences of the Three Src Homology 3 (SH3) Domains from the Adaptor CD2-associated Protein (CD2AP) and Direct Association with Ras and Rab Interactor 3 (RIN3). J Biol Chem 290:25275–25292. doi: 10.1074/jbc.M115.637207

212. Ruggiero A, Aloni E, Korkotian E, Zaltsman Y, Oni-Biton E, Kuperman Y, et al. (2017) Loss of forebrain MTCH2 decreases mitochondria motility and calcium handling and impairs hippocampal-dependent cognitive functions. Sci Rep 7:44401. doi: 10.1038/srep44401

213. Saftig P, Lichtenthaler SF (2015) The alpha secretase ADAM10: A metalloprotease with multiple functions in the brain. Prog Neurobiol 135:1–20. doi: 10.1016/j.pneurobio.2015.10.003

214. Sakae N, Liu C-C, Shinohara M, Frisch-Daiello J, Ma L, Yamazaki Y, et al. (2016) ABCA7 Deficiency Accelerates Amyloid-β Generation and Alzheimer's Neuronal Pathology. J Neurosci 36:3848–59. doi: 10.1523/JNEUROSCI.3757-15.2016

215. Salazar S V, Cox TO, Lee S, Brody AH, Chyung AS, Haas LT, et al. (2018) Alzheimer's Disease Risk Factor Pyk2 Mediates Amyloid-β Induced Synaptic Dysfunction and Loss. J Neurosci 1873–18. doi: 10.1523/JNEUROSCI.1873-18.2018

216. Salazar S V., Strittmatter SM (2017) Cellular prion protein as a receptor for amyloid-β oligomers in Alzheimer's disease. Biochem. Biophys. Res. Commun. 483:1143–1147

217. Sartori M, Mendes T, Desai S, Lasorsa A, Herldean A, Malmanche N, et al. (2018) BIN1 recovers tauopathy-induced long-term memory deficits in mice and interacts with Tau through Thr348 phosphorylation. bioRxiv. doi: https://doi.org/10.1101/462317

218. Sato K, Emi M, Ezura Y, Fujita Y, Takada D, Ishigami T, et al. (2004) Soluble epoxide hydrolase variant (Glu287Arg) modifies plasma total cholesterol and triglyceride phenotype in familial hypercholesterolemia: intrafamilial association study in an eight-generation hyperlipidemic kindred. J Hum Genet 49:29–34. doi: 10.1007/s10038-003-0103-6

219. Satoh K, Abe-Dohmae S, Yokoyama S, St George-Hyslop P, Fraser PE (2015) ATP-binding cassette transporter A7 (ABCA7) loss of function alters Alzheimer amyloid processing. J Biol Chem 290:24152–65. doi: 10.1074/jbc.M115.655076

220. Sayed FA, Telpoukhovskaia M, Kodama L, Li Y, Zhou Y, Le D, et al. (2018) Differential effects of partial and complete loss of TREM2 on microglial injury response and tauopathy. Proc Natl Acad Sci 115:10172–10177. doi: 10.1073/pnas.1811411115

221. Schott JM, Crutch SJ, Carrasquillo MM, Uphill J, Shakespeare TJ, Ryan NS, et al. (2016) Genetic risk factors for the posterior cortical atrophy variant of Alzheimer's disease. Alzheimer's Dement 12:862–871. doi: 10.1016/j.jalz.2016.01.010

222. Sekino S, Kashiwagi Y, Kanazawa H, Takada K, Baba T, Sato S, et al. (2015) The NESH/Abi-3-based WAVE2 complex is functionally distinct from the Abi-1-based WAVE2 complex. Cell Commun Signal 13:41. doi: 10.1186/s12964-015-0119-5

223. Sekiya M, Wang M, Fujisaki N, Sakakibara Y, Quan X, Ehrlich ME, et al. (2018) Integrated biology approach reveals molecular and pathological interactions among Alzheimer's Aβ42, Tau, TREM2, and TYROBP in Drosophila models. Genome Med 10:26. doi: 10.1186/s13073-018-0530-9

224. Serneels L, Van Biervliet J, Craessaerts K, Dejaegere T, Horre K, Van Houtvin T, et al. (2009) -Secretase Heterogeneity in the Aph1 Subunit: Relevance for Alzheimer's Disease. Science (80-) 324:639–642. doi: 10.1126/science.1171176

225. Seshadri S, Fitzpatrick AL, Ikram MA, DeStefano AL, Gudnason V, Boada M, et al. (2010) Genome-wide analysis of genetic loci associated with Alzheimer disease. JAMA 303:1832–40. doi: 10.1001/jama.2010.574

226. Shamah SM, Lin MZ, Goldberg JL, Estrach S, Sahin M, Hu L, et al. (2001) EphA receptors regulate growth cone dynamics through the novel guanine nucleotide exchange factor ephexin. Cell 105:233–44

227. Sharifi R, Morra R, Denise Appel C, Tallis M, Chioza B, Jankevicius G, et al. (2013) Deficiency of terminal ADP-ribose protein glycohydrolase TARG1/C6orf130 in neurodegenerative disease. EMBO J 32:1225–1237. doi: 10.1038/emboj.2013.51

228. Sharma S (2017) Immunomodulation: A definitive role of microRNA-142. Dev Comp Immunol 77:150–156. doi: 10.1016/j.dci.2017.08.001

229. Sharma S, Khadimallah I, Corya AW, Ali YO, Rao X, Liu Y, et al. (2018) Presymptomatic change in microRNAs modulates Tau pathology. Sci Rep 8:9251. doi: 10.1038/s41598-018-27527-6

230. Shen J, Wu J (2015) Nicotinic Cholinergic Mechanisms in Alzheimer's Disease. In: International review of neurobiology. pp 275–292

231. Shen K, Sidik H, Talbot WS (2016) The Rag-Ragulator Complex Regulates Lysosome Function and Phagocytic Flux in Microglia. Cell Rep 14:547–559. doi: 10.1016/j.celrep.2015.12.055

232. Shi Y, Holtzman DM (2018) Interplay between innate immunity and Alzheimer disease: APOE and TREM2 in the spotlight. Nat Rev Immunol 18:759–772. doi: 10.1038/s41577-018-0051-1

233. Shi Y, Yamada K, Liddelow SA, Smith ST, Zhao L, Luo W, et al. (2017) ApoE4 markedly exacerbates tau-mediated neurodegeneration in a mouse model of tauopathy. Nature 549:523–527. doi: 10.1038/nature24016

234. Shulman JM, Chen K, Keenan BT, Chibnik LB, Fleisher A, Thiyyagura P, et al. (2013) Genetic Susceptibility for Alzheimer Disease Neuritic Plaque Pathology. JAMA Neurol 70:1150. doi: 10.1001/jamaneurol.2013.2815

235. Shulman JM, Imboywa S, Giagtzoglou N, Powers MP, Hu Y, Devenport D, et al. (2014) Functional screening in Drosophila identifies Alzheimer's disease susceptibility genes and implicates Tau-mediated mechanisms. Hum Mol Genet 23:870–7. doi: 10.1093/hmg/ddt478

236. Siddiqui SS, Springer SA, Verhagen A, Sundaramurthy V, Alisson-Silva F, Jiang W, et al. (2017) The Alzheimer's disease–protective CD33 splice variant mediates adaptive loss of function via diversion to an intracellular pool. J Biol Chem 292:15312–15320. doi: 10.1074/jbc.M117.799346

237. Sims R, van der Lee SJ, Naj AC, Bellenguez C, Badarinarayan N, Jakobsdottir J, et al. (2017) Rare coding variants in PLCG2, ABI3, and TREM2 implicate microglial-mediated innate immunity in Alzheimer's disease. Nat Genet 49:1373–1384. doi: 10.1038/ng.3916

238. Singh MK, Dadke D, Nicolas E, Serebriiskii IG, Apostolou S, Canutescu A, et al. (2008) A novel Cas family member, HEPL, regulates FAK and cell spreading. Mol Biol Cell 19:1627–36. doi: 10.1091/mbc.E07-09-0953

239. Sottejeau Y, Bretteville A, Cantrelle F-X, Malmanche N, Demiaute F, Mendes T, et al. (2015) Tau phosphorylation regulates the interaction between BIN1's SH3 domain and Tau's proline-rich domain. Acta Neuropathol Commun 3:58. doi: 10.1186/s40478-015-0237-8

240. Steinberg S, Stefansson H, Jonsson T, Johannsdottir H, Ingason A, Helgason H, et al. (2015) Loss-of-function variants in ABCA7 confer risk of Alzheimer's disease. Nat Genet. doi: 10.1038/ng.3246

241. Stephan AB, Tobochnik S, Dibattista M, Wall CM, Reisert J, Zhao H (2011) The Na(+)/Ca(2+) exchanger NCKX4 governs termination and adaptation of the mammalian olfactory response. Nat Neurosci 15:131–7. doi: 10.1038/nn.2943

242. Strappazzon F, Torch S, Trioulier Y, Blot B, Sadoul R, Verna J-M (2007) Survival response-linked Pyk2 activation during potassium depletion-induced apoptosis of cerebellar granule neurons. Mol Cell Neurosci 34:355–65. doi: 10.1016/j.mcn.2006.11.012

243. De Strooper B, Karran E (2016) The Cellular Phase of Alzheimer's Disease. Cell 164:603–15. doi: 10.1016/j.cell.2015.12.056

244. Stucchi R, Plucińska G, Hummel JJA, Zahavi EE, Guerra San Juan I, Klykov O, et al. (2018) Regulation of KIF1A-Driven Dense Core Vesicle Transport: Ca 2+ /CaM Controls DCV Binding and Liprin-α/TANC2 Recruits DCVs to Postsynaptic Sites. Cell Rep 24:685–700. doi: 10.1016/j.celrep.2018.06.071

245. Su W, Hopkins S, Nesser NK, Sopher B, Silvestroni A, Ammanuel S, et al. (2014) The p53 Transcription Factor Modulates Microglia Behavior through MicroRNA-Dependent Regulation of c-Maf. J Immunol 192:358–366. doi: 10.4049/jimmunol.1301397

246. Suárez-Calvet M, Kleinberger G, Araque Caballero MÁ, Brendel M, Rominger A, Alcolea D, et al. (2016) sTREM2 cerebrospinal fluid levels are a potential biomarker for microglia activity in early-stage Alzheimer's disease and associate with neuronal injury markers. EMBO Mol Med 8:466–476. doi: 10.15252/emmm.201506123

247. Sudom A, Talreja S, Danao J, Bragg E, Kegel R, Min X, et al. (2018) Molecular basis for the loss-of-function effects of the Alzheimer's disease–associated R47H variant of the immune receptor TREM2. J Biol Chem 293:12634–12646. doi: 10.1074/jbc.RA118.002352

248. Suh J, Choi SH, Romano DM, Gannon MA, Lesinski AN, Kim DY, et al. (2013) ADAM10 Missense Mutations Potentiate β-Amyloid Accumulation by Impairing Prodomain Chaperone Function. Neuron 80:385–401. doi: 10.1016/j.neuron.2013.08.035

249. Sun X, Becker M, Pankow K, Krause E, Ringling M, Beyermann M, et al. (2008) Catabolic attacks of membrane-bound angiotensin-converting enzyme on the N-terminal part of species-specific amyloid-β peptides. Eur J Pharmacol 588:18–25. doi: 10.1016/j.ejphar.2008.03.058

250. Sze C-I, Su M, Pugazhenthi S, Jambal P, Hsu L-J, Heath J, et al. (2004) Down-regulation of WW Domain-containing Oxidoreductase Induces Tau Phosphorylation *in Vitro*. J Biol Chem 279:30498–30506. doi: 10.1074/jbc.M401399200

251. Tao Q-Q, Liu Z-J, Sun Y-M, Li H-L, Yang P, Liu D-S, et al. (2017) Decreased gene expression of CD2AP in Chinese patients with sporadic Alzheimer's disease. Neurobiol Aging 56:212.e5–212.e10. doi: 10.1016/j.neurobiolaging.2017.03.013

252. Thomas RS, Henson A, Gerrish A, Jones L, Williams J, Kidd EJ (2016) Decreasing the expression of PICALM reduces endocytosis and the activity of β-secretase: implications for Alzheimer's disease. BMC Neurosci 17:50. doi: 10.1186/s12868-016-0288-1

253. Tian Y, Chang JC, Fan EY, Flajolet M, Greengard P (2013) Adaptor complex AP2/PICALM, through interaction with LC3, targets Alzheimer's APP-CTF for terminal degradation via autophagy. Proc Natl Acad Sci 110:17071–17076. doi: 10.1073/pnas.1315110110

254. Tomioka M, Toda Y, Mañucat NB, Akatsu H, Fukumoto M, Kono N, et al. (2017) Lysophosphatidylcholine export by human ABCA7. Biochim Biophys Acta - Mol Cell Biol Lipids 1862:658–665. doi: 10.1016/j.bbalip.2017.03.012

255. Toropygin IY, Kugaevskaya E V, Mirgorodskaya OA, Elisseeva YE, Kozmin YP, Popov IA, et al. (2008) The N-domain of angiotensin-converting enzyme specifically hydrolyzes the Arg-5-His-6 bond of Alzheimer's Abeta-(1-16) peptide and its isoAsp-7 analogue with different efficiency as evidenced by quantitative matrix-assisted laser desorption/ionization time-of-flight mass spectrometry. Rapid Commun Mass Spectrom 22:231–9. doi: 10.1002/rcm.3357

256. Treusch S, Hamamichi S, Goodman JL, Matlack KES, Chung CY, Baru V, et al. (2011) Functional Links Between A Toxicity, Endocytic Trafficking, and Alzheimer's Disease Risk Factors in Yeast. Science (80-) 334:1241–1245. doi: 10.1126/science.1213210

257. Troy CM, Rabacchi SA, Xu Z, Maroney AC, Connors TJ, Shelanski ML, et al. (2001) beta-Amyloid-induced neuronal apoptosis requires c-Jun N-terminal kinase activation. J Neurochem 77:157–64

258. Tsui CC, Pierchala BA (2008) CD2AP and Cbl-3/Cbl-c Constitute a Critical Checkpoint in the Regulation of Ret Signal Transduction. J Neurosci 28:8789–8800. doi: 10.1523/JNEUROSCI.2738-08.2008

259. Tzioras M, Davies C, Newman A, Jackson R, Spires-Jones T (2018) Invited Review: APOE at the interface of inflammation, neurodegeneration and pathological protein spread in Alzheimer's disease. Neuropathol Appl Neurobiol. doi: 10.1111/nan.12529

260. Ubelmann F, Burrinha T, Salavessa L, Gomes R, Ferreira C, Moreno N, et al. (2017) Bin1 and CD2AP polarise the endocytic generation of beta-amyloid. EMBO Rep 18:102–122. doi: 10.15252/embr.201642738

261. Ulland TK, Colonna M (2018) TREM2 — a key player in microglial biology and Alzheimer disease. Nat Rev Neurol 14:667–675. doi: 10.1038/s41582-018-0072-1

262. Ulrich JD, Finn M, Wang Y, Shen A, Mahan TE, Jiang H, et al. (2014) Altered microglial response to Aβ plaques in APPPS1-21 mice heterozygous for TREM2. Mol Neurodegener 9:20. doi: 10.1186/1750-1326-9-20

263. Vardarajan BN, Zhang Y, Lee JH, Cheng R, Bohm C, Ghani M, et al. (2015) Coding mutations in *SORL 1* and Alzheimer disease. Ann Neurol 77:215–227. doi: 10.1002/ana.24305

264. Vargas LM, Leal N, Estrada LD, González A, Serrano F, Araya K, et al. (2014) EphA4 activation of c-Abl mediates synaptic loss and LTP blockade caused by amyloid-β oligomers. PLoS One 9:e92309. doi: 10.1371/journal.pone.0092309

265. Del Villar K, Miller CA (2004) Down-regulation of DENN/MADD, a TNF receptor binding protein, correlates with neuronal cell death in Alzheimer's disease brain and hippocampal neurons. Proc Natl Acad Sci U S A 101:4210–5. doi: 10.1073/pnas.0307349101

266. Villegas-Llerena C, Phillips A, Garcia-Reitboeck P, Hardy J, Pocock JM (2016) Microglial genes regulating neuroinflammation in the progression of Alzheimer's disease. Curr Opin Neurobiol 36:74–81. doi: 10.1016/j.conb.2015.10.004

267. Walter S, Jumpertz T, Hüttenrauch M, Ogorek I, Gerber H, Storck SE, et al. (2018) The metalloprotease ADAMTS4 generates N-truncated Aβ4–x species and marks oligodendrocytes as a source of amyloidogenic peptides in Alzheimer's disease. Acta Neuropathol. doi: 10.1007/s00401-018-1929-5

268. Wang Y -b., Wang J -j., Wang S -h., Liu S-S, Cao J -y., Li X -m., et al. (2012) Adaptor Protein APPL1 Couples Synaptic NMDA Receptor with Neuronal Prosurvival Phosphatidylinositol 3-Kinase/Akt Pathway. J Neurosci 32:11919–11929. doi: 10.1523/JNEUROSCI.3852-11.2012

269. Wang Y, Cella M, Mallinson K, Ulrich JD, Young KL, Robinette ML, et al. (2015) TREM2 Lipid Sensing Sustains the Microglial Response in an Alzheimer's Disease Model. Cell 160:1061–1071. doi: 10.1016/j.cell.2015.01.049

270. White AO, Kramár EA, López AJ, Kwapis JL, Doan J, Saldana D, et al. (2016) BDNF rescues BAF53b-dependent synaptic plasticity and cocaine-associated memory in the nucleus accumbens. Nat Commun 7:11725. doi: 10.1038/ncomms11725

271. Willnow TE, Andersen OM (2013) Sorting receptor SORLA--a trafficking path to avoid Alzheimer disease. J Cell Sci 126:2751–60. doi: 10.1242/jcs.125393

272. Wilson MR, Zoubeidi A (2017) Clusterin as a therapeutic target. Expert Opin Ther Targets 21:201–213. doi: 10.1080/14728222.2017.1267142

273. Wixler V, Laplantine E, Geerts D, Sonnenberg A, Petersohn D, Eckes B, et al. (1999) Identification of novel interaction partners for the conserved membrane proximal region of alpha-integrin cytoplasmic domains. FEBS Lett 445:351–5

274. Wojtas AM, Kang SS, Olley BM, Gatherer M, Shinohara M, Lozano PA, et al. (2017) Loss of clusterin shifts amyloid deposition to the cerebrovasculature via disruption of perivascular drainage pathways. Proc Natl Acad Sci 114:E6962–E6971. doi: 10.1073/pnas.1701137114

275. Wong W, Scott JD (2004) AKAP signalling complexes: focal points in space and time. Nat Rev Mol Cell Biol 5:959–970. doi: 10.1038/nrm1527

276. Wright S, Malinin NL, Powell KA, Yednock T, Rydel RE, Griswold-Prenner I (2007) Alpha2beta1 and alphaVbeta1 integrin signaling pathways mediate amyloid-beta-induced neurotoxicity. Neurobiol Aging 28:226–37. doi: 10.1016/j.neurobiolaging.2005.12.002

277. Wu H-F, Chen Y-J, Wu S-Z, Lee C-W, Chen I-T, Lee Y-C, et al. (2017) Soluble Epoxide Hydrolase Inhibitor and 14,15-Epoxyeicosatrienoic Acid-Facilitated Long-Term Potentiation through cAMP and CaMKII in the Hippocampus. Neural Plast 2017:1–14. doi: 10.1155/2017/3467805

278. Xiang X, Werner G, Bohrmann B, Liesz A, Mazaheri F, Capell A, et al. (2016) TREM2 deficiency reduces the efficacy of immunotherapeutic amyloid clearance. EMBO Mol Med 8:992–1004. doi: 10.15252/emmm.201606370

279. Xiang Z, Haroutunian V, Ho L, Purohit D, Pasinetti GM (2006) Microglia activation in the brain as inflammatory biomarker of Alzheimer's disease neuropathology and clinical dementia. Dis Markers 22:95–102

280. Xiao Q, Gil S-C, Yan P, Wang Y, Han S, Gonzales E, et al. (2012) Role of Phosphatidylinositol Clathrin Assembly Lymphoid-Myeloid Leukemia (PICALM) in Intracellular Amyloid Precursor Protein (APP) Processing and Amyloid Plaque Pathogenesis. J Biol Chem 287:21279–21289. doi: 10.1074/jbc.M111.338376

281. Xu W, Tan L, Yu J-T (2015) The Role of PICALM in Alzheimer's Disease. Mol Neurobiol 52:399–413. doi: 10.1007/s12035-014-8878-3

282. Yamazaki T, Masuda J, Omori T, Usui R, Akiyama H, Maru Y (2009) EphA1 interacts with integrin-linked kinase and regulates cell morphology and motility. J Cell Sci 122:243–255. doi: 10.1242/jcs.036467

283. Yan X, Nykänen N-P, Brunello CA, Haapasalo A, Hiltunen M, Uronen R-L, et al. (2016) FRMD4A–cytohesin signaling modulates the cellular release of tau. J Cell Sci 129:2003–2015. doi: 10.1242/jcs.180745

284. Yerbury JJ, Poon S, Meehan S, Thompson B, Kumita JR, Dobson CM, et al. (2007) The extracellular chaperone clusterin influences amyloid formation and toxicity by interacting with prefibrillar structures. FASEB J 21:2312–2322. doi: 10.1096/fj.06-7986com

285. Yokoyama K, Tezuka T, Kotani M, Nakazawa T, Hoshina N, Shimoda Y, et al. (2011) NYAP: a phosphoprotein family that links PI3K to WAVE1 signalling in neurons. EMBO J 30:4739–4754. doi: 10.1038/emboj.2011.348

286. Yoshino Y, Yamazaki K, Ozaki Y, Sao T, Yoshida T, Mori T, et al. (2017) INPP5D mRNA Expression and Cognitive Decline in Japanese Alzheimer's Disease Subjects. J Alzheimer's Dis 58:687–694. doi: 10.3233/JAD-161211

287. Yu L, Chibnik LB, Srivastava GP, Pochet N, Yang J, Xu J, et al. (2015) Association of Brain DNA Methylation in *SORL1* , *ABCA7* , *HLA-DRB5* , *SLC24A4* , and *BIN1* With Pathological Diagnosis of Alzheimer Disease. JAMA Neurol 72:15. doi: 10.1001/jamaneurol.2014.3049

288. Zelcer N, Khanlou N, Clare R, Jiang Q, Reed-Geaghan EG, Landreth GE, et al. (2007) Attenuation of neuroinflammation and Alzheimer's disease pathology by liver x receptors. Proc Natl Acad Sci 104:10601–10606. doi: 10.1073/pnas.0701096104

289. Zhang Y, Sloan SA, Clarke LE, Caneda C, Plaza CA, Blumenthal PD, et al. (2016) Purification and Characterization of Progenitor and Mature Human Astrocytes Reveals Transcriptional and Functional Differences with Mouse. Neuron 89:37–53. doi: 10.1016/j.neuron.2015.11.013

290. Zhang Z-G, Li Y, Ng CT, Song Y-Q (2015) Inflammation in Alzheimer's Disease and Molecular Genetics: Recent Update. Arch Immunol Ther Exp (Warsz) 63:333–344. doi: 10.1007/s00005-015-0351-0

291. Zhao J, Bruck S, Cemerski S, Zhang L, Butler B, Dani A, et al. (2013) CD2AP links cortactin and capping protein at the cell periphery to facilitate formation of lamellipodia. Mol Cell Biol 33:38–47. doi: 10.1128/MCB.00734-12

292. Zhao Q-F, Wan Y, Wang H-F, Sun F-R, Hao X-K, Tan M-S, et al. (2016) ABCA7 Genotypes Confer Alzheimer's Disease Risk by Modulating Amyloid-β Pathology. J Alzheimer's Dis 52:693–703. doi: 10.3233/JAD-151005

293. Zhao X, Kotilinek LA, Smith B, Hlynialuk C, Zahs K, Ramsden M, et al. (2016) Caspase-2 cleavage of tau reversibly impairs memory. Nat Med 22:1268–1276. doi: 10.1038/nm.4199

294. Zhao Z, Sagare AP, Ma Q, Halliday MR, Kong P, Kisler K, et al. (2015) Central role for PICALM in amyloid-β blood-brain barrier transcytosis and clearance. Nat Neurosci 18:978–87. doi: 10.1038/nn.4025

295. Zheng H, Jia L, Liu C-C, Rong Z, Zhong L, Yang L, et al. (2017) TREM2 Promotes Microglial Survival by Activating Wnt/β-Catenin Pathway. J Neurosci 37:1772–1784. doi: 10.1523/JNEUROSCI.2459-16.2017

296. Zheng H, Liu C-C, Atagi Y, Chen X-F, Jia L, Yang L, et al. (2016) Opposing roles of the triggering receptor expressed on myeloid cells 2 and triggering receptor expressed on myeloid cells-like transcript 2 in microglia activation. Neurobiol Aging 42:132–141. doi: 10.1016/j.neurobiolaging.2016.03.004

297. Zhong L, Wang Z, Wang D, Wang Z, Martens YA, Wu L, et al. (2018) Amyloid-beta modulates microglial responses by binding to the triggering receptor expressed on myeloid cells 2 (TREM2). Mol Neurodegener 13:15. doi: 10.1186/s13024-018-0247-7

298. Zhou T, Ma Y, Tang J, Guo F, Dong M, Wei Q (2018) Modulation of *IGF1R* Signaling Pathway by *GIGYF1* in High Glucose-Induced SHSY-5Y Cells. DNA Cell Biol 37:1044–1054. doi: 10.1089/dna.2018.4336

299. Zhou Y, Hayashi I, Wong J, Tugusheva K, Renger JJ, Zerbinatti C (2014) Intracellular Clusterin Interacts with Brain Isoforms of the Bridging Integrator 1 and with the Microtubule-Associated Protein Tau in Alzheimer's Disease. PLoS One 9:e103187. doi: 10.1371/journal.pone.0103187

300. Zhu X-C, Wang H-F, Jiang T, Lu H, Tan M-S, Tan C-C, et al. (2017) Effect of CR1 Genetic Variants on Cerebrospinal Fluid and Neuroimaging Biomarkers in Healthy, Mild Cognitive Impairment and Alzheimer's Disease Cohorts. Mol Neurobiol 54:551–562. doi: 10.1007/s12035-015-9638-8

301. Zhu X-C, Yu J-T, Jiang T, Wang P, Cao L, Tan L (2015) CR1 in Alzheimer's Disease. Mol Neurobiol 51:753–765. doi: 10.1007/s12035-014-8723-8

302. Zlokovic B V, Martel CL, Matsubara E, McComb JG, Zheng G, McCluskey RT, et al. (1996) Glycoprotein 330/megalin: probable role in receptor-mediated transport of apolipoprotein J alone and in a complex with Alzheimer disease amyloid beta at the blood-brain and blood-cerebrospinal fluid barriers. Proc Natl Acad Sci U S A 93:4229–34
